# EM-Wave Biosensors: A Review of RF, Microwave, mm-Wave and Optical Sensing

**DOI:** 10.3390/s19051013

**Published:** 2019-02-27

**Authors:** Parikha Mehrotra, Baibhab Chatterjee, Shreyas Sen

**Affiliations:** School of Electrical and Computer Engineering, Purdue University, West Lafayette, IN 47906, USA; bchatte@purdue.edu (B.C.); shreyas@purdue.edu (S.S.)

**Keywords:** permittivity, polarization, optical, microwave, millimeter, RF, biosensor, terahertz, surface plasmon resonance, split ring resonator, photoplethysmography

## Abstract

This article presents a broad review on optical, radio-frequency (RF), microwave (MW), millimeter wave (mmW) and terahertz (THz) biosensors. Biomatter-wave interaction modalities are considered over a wide range of frequencies and applications such as detection of cancer biomarkers, biotin, neurotransmitters and heart rate are presented in detail. By treating biological tissue as a dielectric substance, having a unique dielectric signature, it can be characterized by frequency dependent parameters such as permittivity and conductivity. By observing the unique permittivity spectrum, cancerous cells can be distinguished from healthy ones or by measuring the changes in permittivity, concentration of medically relevant biomolecules such as glucose, neurotransmitters, vitamins and proteins, ailments and abnormalities can be detected. In case of optical biosensors, any change in permittivity is transduced to a change in optical properties such as photoluminescence, interference pattern, reflection intensity and reflection angle through techniques like quantum dots, interferometry, surface enhanced raman scattering or surface plasmon resonance. Conversely, in case of RF, MW, mmW and THz biosensors, capacitive sensing is most commonly employed where changes in permittivity are reflected as changes in capacitance, through components like interdigitated electrodes, resonators and microstrip structures. In this paper, interactions of EM waves with biomatter are considered, with an emphasis on a clear demarcation of various modalities, their underlying principles and applications.

## 1. Introduction

A biosensor is a detection device which converts a biological response into a measurable signal. Past developments in biosensing have reaped huge benefits in the medical domain [1]. It offers the ability for detection of several types of cancers, vital signs such as heart rate, detection of biomolecules such as DNA and proteins for detection of genetic disorders, and detection of glucose for diabetes. Biomatter is considered a dielectric substance [2], exhibiting conductivity on application of electric field. This dielectric property is measured in terms of permittivity and is utilized for sensing.

**Figure 1:**
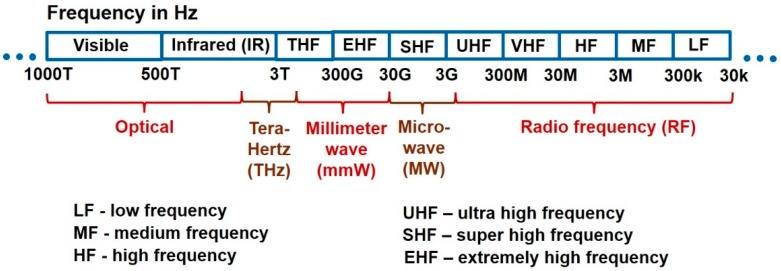
EM spectrum showing the radio frequency, millimeter wave, microwave, terahertz, infrared and visible band ranges [3].

In this paper, we review of the methodologies employed in electromagnetic (EM) wave biosensors, namely RF, MW, mmW, THz and optical biosensors. Figure 1 shows the different frequency bands in the EM spectrum and their nomenclature according to the IEEE Std [3]. The crux of EM wave sensing is observing the unique permittivity spectrum of cells and tissues to identify abnormalities (e.g., cancerous cells will have a different permittivity spectrum from healthy cells) or measuring the changes in permittivity to quantify the concentration of biomolecules (e.g., glucose, biotin, serotonin, prostate specific antigen) whose presence in high quantities may imply an ailment or disorder. In case of optical biosensors (a subset of EM wave biosensors), instead of measuring in terms of permittivity, we sense the optical properties of biomatter, for instance the amount of reflected light or changes in optical properties. The popular techniques employed by optical biosensors include quantum dots, photonic crystals, fluorescence, ellipsometry, surface plasmon resonance, laser Doppler flowmetry, surface enhanced Raman scattering and backscattering. RF/mmW/MW/THz biosensors employ capacitive sensing (measuring permittivity in terms of capacitance or scattering parameters) using interdigitated capacitors, resonators and microstrip structures. In current scenario, though electrochemical biosensors dominate the commercial market, EM wave biosensors are attracting a lot of attention due to benefits such as being minimally invasive, label-free and cost effective.

This paper presents a broad review on biosensors employing EM waves, in particular RF/MW/mmW/THz and optical biosensors. The interaction modalities of EM waves with biomatter encompassing the frequency range of 3 Hz–790 THz is discussed along with its applications in the medical domain. Section 1 elaborates upon the dielectric properties of biomatter along with the phenomenons taking place inside a living organism on interaction with EM waves, Section 2 builds insight regarding the choice of frequency range for a particular sensing application, followed by Section 3 which lists the commercially available medical biosensors and point of care testing devices. Section 4 and Section 5 enumerate the biomatter-wave interaction modalities of RF/mmW/MW/THz and optical biosensors respectively. Section 6 discusses about the safetly aspects of exposing EM waves on living organisms followed by conclusions in Section 7.

### 1.1. Dielectric Properties and Polarization

**Figure 2:**
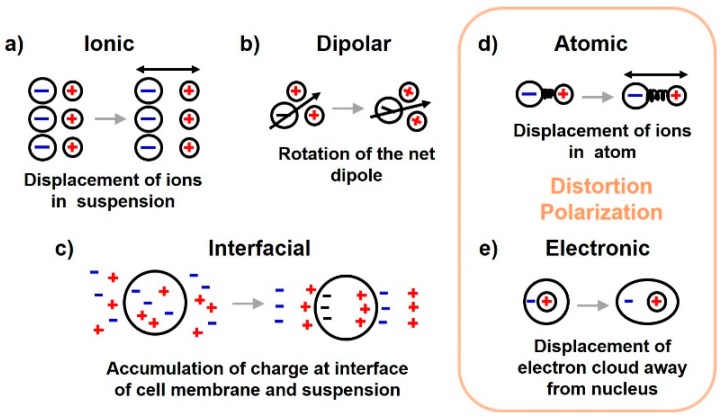
Application of electric field to a dielectric, such as biomatter, leads to different types of polarization mechanisms: ionic, interfacial dipolar, atomic and electronic polarizations.

Dielectrics, unlike conductors, do not possess free electrons for conduction. However, they exhibit conduction properties under an applied field. On application of electric field, the positive charges in the dielectric get displaced in the direction of electric field and negative charges get displaced in the opposite direction. The conduction properties are attributed to this net charge separation. *Polarization* is this ordering in space of electrical charges in response to an external alternating electric field and can be classified into ionic, interfacial, dipolar and distortion polarization [4] as shown in Figure 2. Distortion polarization can be further classified into electronic and atomic polarizations.

**Figure 3:**
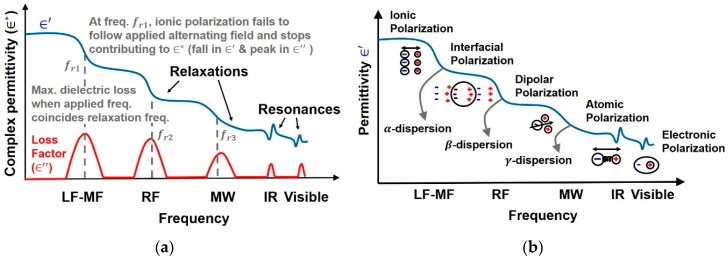
(**a**) The behavior observed when an alternating field of increasing frequency is applied to a dielectric. Relaxation phenomenon is observed as a drop in real part of permittivity whereas resonance is observed as a trough and peak in succession. Both resonance and relaxation phenomenon cause loss in electromagnetic energy. (**b**) The order of polarization and relaxation mechanisms observed when an alternating electric field of increasing frequency is applied to biological matter. Ionic diffusion is observed first, followed by interfacial and dipolar relaxations, followed by atomic and electronic resonances.

There is a fundamental difference in dynamics of the mechanisms of distortion polarization compared to other polarizations with respect to the changing forces. In distortion polarization (electronic and atomic), the electric field tries to change the distance between the charges involved and in response a restoring force acts. In classical terms [5] it behaves as a resonator and is characterized by a *resonance frequency*. It can be seen as a peak in imaginary part of permittivity ($\in^{\prime \prime }$) and a peak followed by a trough in real part of permittivity ($\in^{\prime }$) in Figure 3. In case of ionic, interfacial and dipolar polarizations there is no direct mechanical force pulling the dipoles back to random orientation and is characterized by *relaxation frequency*, seen as a fall in $\in^{\prime }$ and a peak in $\in^{\prime \prime }$.

As mentioned above, five types of polarization mechanisms are observed when a dielectric is subjected to an alternating field of increasing frequency as shown in Figure 3b. On subjecting the dielectric with a field of increasing frequency, first polarization of ions is observed, followed by polarization of molecules and finally atomic polarization is observed at very high frequencies. Their detailed description is provided next. (a) *Ionic polarization* is the displacement of ions in an ionic aqueous medium from their equilibrium position on application of electric field. (b) *Electronic, atomic and dipolar polarizations* occur due to charges locally bound in atoms and molecules, however, charge carriers may also be present that are capable of migrating to some distance via bulk of the material through diffusion or hopping when a low frequency field is applied. Their motion is impeded at an interface and in case of tissues, it occurs at the cell membrane. This type of polarization is referred to as *space-charge* or *interfacial* or *Maxwell-Wagner polarization*. (c) In the case of polar molecules (already having a net permanent dipole) such as water, the molecular dipoles which are otherwise randomly distributed get rotated in the direction of electric field giving a net polarization in that direction. This is known as *dipolar* or *oriented polarization*. (d) *Atomic polarization* occurs in the IR band when adjacent positive and negative ions in an atom stretch under an applied field. (e) Finally, displacement of electrons on application of electric field with respect to the center of its nucleus, gives rise to *electronic polarization* which resonates at frequencies in the visible band.

Let us now look at the mathematical representation of EM-wave interaction with dielectrics. We know that application of electric field leads to displacement of charges in a dielectric, inducing dipoles. The average dipole moment (μ), with charge *q* and charge separation $x_{i}$ is given by Equation (1):

(1) $$\mu =qx_{i}$$

Polarization density (P) [4] is the sum of all such dipole moments in a volume δv with N dipoles (Equation (2)).

(2) $$P=\frac{\sum_{i=1}^{N}qx_{i}}{\delta v}$$

As given by Maxwell [6], *P* is the density of permanent and induced dipole moments in a system, D is the electric charge density vector also known as displacement field and accounts for the total charge in the system and the difference in vectors (*D* & *P*) accounts for the remaining free charges in the dielectric, given as Equation (3):

(3) $$D=\epsilon_{0}E+P$$

Alternatively, the displacement density can be written as Equation (4):

(4) $$D=\epsilon^{\prime }\epsilon_{0}E,$$

where $\epsilon_{0}$ denotes permittivity of free space and $\epsilon^{\prime }$ is relative permittivity. Combining Equations (3) and (4), we get Equation (5):

(5) $$P=(\epsilon^{\prime }-1)\epsilon_{0}E$$

Now, to obtain the dependence of permittivity $\left(\epsilon^{\prime }\right)$ on each type of polarization, we consider the polarization field (*P*) to be made of $N^{\prime }$ individual dipole moments (μ), given as Equation (6):

(6) $$P=\;\mu N^{\prime }$$

The dipole moment can be assumed linear to the local field $E^{\prime }$ experienced by the dipole [6] (induced dipoles give rise to an induced field which opposes E, resulting in net field $E^{\prime }$), given as Equation (7):

(7) $$\mu =\alpha_{t}E^{\prime },$$

where $\alpha_{t}$ is the proportionality constant known as polarizability. Different polarization mechanisms contribute towards the total polarizability $\left(\alpha_{t}\right)$ of the dielectric, given as Equation (8):

(8) $$\alpha_{t}=\alpha_{e}+\alpha_{a}+\alpha_{d}+\alpha_{MW}+\alpha_{i},$$

where $\alpha_{e},\;\alpha_{a},\alpha_{d},\alpha_{MW}\;\mathrm{and}\;\alpha_{i}$ are the electronic, atomic, dipolar, interfacial and ionic polarizabilities respectively. Combining Equations (5)–(7), we obtain Equation (9):

(9) $$(\epsilon^{\prime }-1)\epsilon_{0}E=\alpha_{t}E^{\prime }N^{\prime }$$

Equation (9) relates the macroscopic quantities $\epsilon^{\prime }\;\&\;E$ to the molecular parameters $E^{\prime }\;\&\;N^{\prime }$. We can note from Equation (9) that permittivity $\left(\epsilon^{\prime }\right)$ depends on the number of mechanisms contributing towards total polarization (through $\alpha_{t}$). As discussed later in Section 1.3, at low frequencies all the mechanisms (ionic, interfacial, dipolar, distortion polarization) contribute towards total polarization and permittivity is maximum (Figure 3). With increase in frequency, the polarizations/dipoles which are unable to align with the fast changing electric field, are dropped, no longer contributing towards polarizability ($\alpha_{t}$) resulting in a fall in permittivity. As shown in Figure 3, ionic polarization is the first one to be dropped due to its inability to keep up with the increase in frequency of the applied alternating field, followed by interfacial and dipolar polarizations. Permittivity also depends on the number of dipoles in the given volume. Thus, increasing the volume, increases $N^{\prime }$ and consequenctly $\epsilon^{\prime }$. Hence there is an opportunity to utilize this phenomenon to measure the concentration of biomolecules by observing the changes in permittivity, as done in glucose [5], heparin [7] and melanoma [8] biosensors.

### 1.2. Permittivity

Conduction of charges in a dielectric gives rise to conduction currents, while displacement of charges due to polarization leads to displacement currents. To account for loss of electromagnetic energy in a dielectric attributed to polarization and conduction currents [6], permittivity attains a complex form $\epsilon^{*}$, where the imaginary part is called the loss factor (Equation (10)):

(10) $$\epsilon^{*}=\epsilon^{\prime }-j\epsilon^{\prime \prime },$$

$\epsilon^{\prime }$ is the real part of permittivity and $\epsilon^{\prime \prime }$ is the loss factor due to conduction and polarization losses. The effective loss factor is given by Equation (11):

(11) $$\epsilon_{eff}^{\prime \prime }\left(\omega \right)=\epsilon_{d}^{\prime \prime }\left(\omega \right)+\epsilon_{e}^{\prime \prime }\left(\omega \right)+\epsilon_{a}^{\prime \prime }\left(\omega \right)+\epsilon_{MW}^{\prime \prime }\left(\omega \right)+\epsilon_{i}^{\prime \prime }\left(\omega \right)+\frac{\sigma }{\epsilon_{0}}\omega$$

$\frac{\sigma }{\epsilon_{0}}\omega$ is the conduction loss [6], where σ denotes conductivity of charges through the dielectric and ω is frequency. The rest of the factors represent polarization losses. $\epsilon_{d}^{\prime \prime }$, $\;\epsilon_{e}^{\prime \prime },\;\epsilon_{a}^{\prime \prime },\epsilon_{MW}^{\prime \prime }$ and $\epsilon_{i}^{\prime \prime }$ denote dielectric loss factors due to dipolar, electronic, atomic, interfacial and ionic polarizations respectively and are a function of frequency.

All EM wave sensors rely on detecting dielectric properties, i.e., permittivity, however, optical biosensors sense optical properties (which are a transduced version of permittivity) and not permittivity directly, hence we limit the rest of the introduction to frequency region from radio frequency upto few terahertz.

### 1.3. Debye Equations

As mentioned earlier in Section 1.1, dipoles try to align in the direction of the applied field. As the field direction is reversed, the alignment of the dipole changes. The time taken by the charges to catch up with the changing field which is known as *relaxation time*. Thus, as the frequency increases, the charges get lesser time to align and eventually fail to keep up. This frequency is known as *relaxation frequency*. Alternatively, we can say that polarization lags applied field, as established by Debye [6]. If we consider an electric field (*E*), given by Equation (12):

(12) $$E=E_{max}\sin\omega t$$

Then, *P* is given by Equation (13):

(13) $$P=P_{max}\sin\left(\omega t-\varphi \right)$$

where $E_{max}$ and $P_{max}$ are constants, and φ accounts for the phase difference between *E* and *P* vectors. The resulting current is given by Equation (14):

(14) $$\frac{\partial P}{\partial t}=\omega P_{max}\cos\left(\omega t-\varphi \right)$$

and the average power dissipated into the dielectric is formulated as Equation (15) [6]:

(15) $$P_{av}=\frac{1}{2}E_{max}P_{max}\;\omega \sin\varphi$$

It can be noted from Equation (15) that if the phase difference (φ) is zero, no power is dissipated in the dielectric and energy is stored like in a capacitor. Furthermore, Debye [6] showed the dependence of permittivity on frequency and relaxation times (τ) to be as given in Equation (16):

(16) $$\epsilon^{*}=\epsilon^{\prime }-j\epsilon^{\prime \prime }=\epsilon_{\infty }+\frac{\epsilon_{s}-\epsilon_{\infty }}{1+j\omega \tau }\;$$

where $\epsilon_{s}$ and $\epsilon_{\infty }$ are dielectric constants at d.c. and at very high frequencies respectively. Separating the real and imaginary parts of permittivity, we get Equations (17) and (18):

(17) $$\epsilon^{\prime }=\epsilon_{\infty }+\frac{\epsilon_{s}-\epsilon_{\infty }}{1+\omega^{2}\tau^{2}}$$

(18) $$\epsilon^{\prime \prime }=\frac{(\epsilon_{s}-\epsilon_{\infty })\omega \tau }{1+\omega^{2}\tau^{2}}$$

Equation (16) accounts for just one type of polarization mechanism (e.g., dipolar polarization) having relaxation time τ. In general, each polarization mechanism will have its own relaxation time and will get added in Equation (16) to give Equation (19):

(19) $$\epsilon^{*}=\epsilon_{\infty }+\frac{\epsilon_{s}-\epsilon_{\infty }}{1+j\omega \tau_{i}}+\frac{\epsilon_{s}-\epsilon_{\infty }}{1+j\omega \tau_{MW}}+\frac{\epsilon_{s}-\epsilon_{\infty }}{1+j\omega \tau_{d}}+\frac{\epsilon_{s}-\epsilon_{\infty }}{1+j\omega \tau_{a}}+\frac{\epsilon_{s}-\epsilon_{\infty }}{1+j\omega \tau_{e}}\;$$

where $\tau_{i},\;\tau_{MW},\;\tau_{d}$, $\tau_{a}$ and $\tau_{e}$ represent the relaxation times of ionic, interfacial, dipolar, atomic and electronic polarizations respectively. The inverse of relaxation time is relaxation frequency, shown in Figure 3 as a drop in permittiivty, given by Equation (20):

(20) $$f_{r}=\frac{1}{\tau },$$

Equation (18) indicates that the dielectric loss is maximum when frequency of the applied field coincides with the relaxation frequency for a given polarization (i.e., ωτ=1). For instance, the $f_{r}$ of water lies in the microwave region and this concept is exploited in ovens, where the applied frequency matches $f_{r}$ and loss in electromagnetic energy at relaxation frequency is used for heating.

### 1.4. Polarization in Tissues

Debye’s equations provide insight into the mechanism behind permittivity variation with frequency. At low frequencies, dipoles have ample time to follow the varying applied field, hence phase difference between *E* and *P* vectors (φ) is zero, implying zero dielctric loss and maximum permittivity. As the frequency increases, the dipoles are unable to fully restore their original positions during field reversals and *P* lags *E*, leading to loss in permittivity. When applied frequency coincides with the relaxation frequency, polarization fails to keep up with the fast changing electric field and drops out, and stops contributing, seen as a fall in permittivity. This frequency dependence of permittivity is known as *dispersion* [9]. Figure 3b shows the general trend of dispersions with increase in applied frequency on biomatter [10].

The first dispersion, named as α-dispersion occurs as a result of ionic relaxation occurring in the kHz range, in the cell suspension medium. Polarizability is due to contributions from all mechanisms giving maximum permittivity, however, at α-dispersion, the ionic polarization fails to follow the electric field leading to high dielectric loss seen as a dip in real permittivity and a peak in loss factor. The second dispersion known as β-dispersion, in the MHz region is associated with interfacial relaxation at the cell membrane between charges inside cytoplasm and outside the cell membrane in the suspension medium [10]. At GHz frequencies, γ-dispersion occurs as a result of dipolar relaxation of water and several proteins (major constituents of cell cytoplasm). In this region, dipolar polarization stops contributing towards polarizability, and at dipolar relaxation frequency, dielectric loss peaks up while the real permittivity falls.

**Figure 4:**
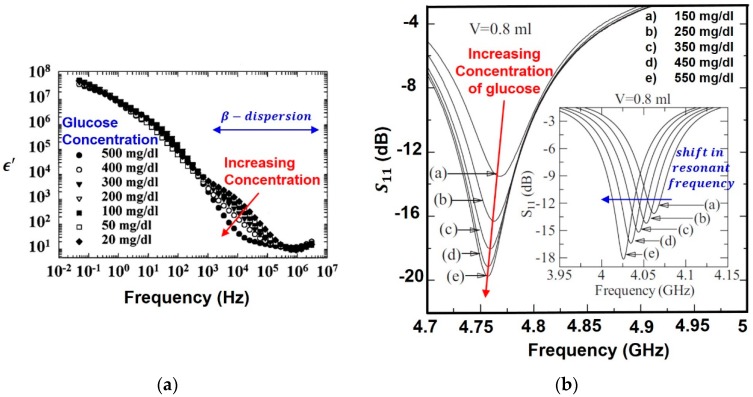
(**a**) Broadband detection of glucose concentration by observing permittivity over a wide frequency range [15]. (**b**) Narrowband detection of glucose concentration using resonator-based capacitive sensing and observing change in reflection coefficient magnitude and resonance frequency [16].

In summary, the variation of permittivity as a function of frequency is known as dispersion. A sharp drop in permittivty occurs at relaxation frequency marked by a loss in electromagnetic energy which occurs when a particular polarization fails to keep up with the fast changing electric field. Furthermore, this permittivity spectrum provides a unique dielctric signature and can be utilized for sensing purpose. The biological target can be biomolecules, like proteins, DNA, biomarkers, pathogenic organisms, hormones, or other medically relevant analytes like glucose, medical parameters like pulse, heart-beat, etc. Biosensing applications include identification of these biological targets such as cancer cells (hepatoma liver cancer cells, skin cancer melanoma cells, blood cancer lymphoma cells), detection of biomarkers [11] (prostate specific antigen (PSA) for prostate cancer, Human epididymis protein 4 (HE4) for ovarian cancer), detection of vital signs (respiration and heart rate) and detection of biomolecules (glucose, cortisol, heparin). For instance, in glucose biosensors, as shown in Figure 4a, change in concentration of glucose on the detection surface leads to change in permittivity and shift in relaxation frequency which is detected. In case of biomarker detection, biomarkers are captured at the detection surface leading to a change in permittivity which is sensed. In general, permittivity is different for different biological tissues, providing unique dielectric signatures for all. Dead [12] and tumor [13] cells differ in their cytoplasmic contents from living and healthy cells due to the presence of pores and blisters [14], providing a permittivity contrast. By directly measuring the unique permittivity curves of cells and tissues or by indirectly measuring the changes in permittivity, we can perform the aforementioned biosensing applications.

There are two prominent ways of observing changes in permittivity, either over a wide range of frequencies (broadband) or over a small frequency band (narrowband). Figure 4a [15] shows the broadband permittivity spectrum of glucose (Section 4.2.3). As shown later in Section 4.2.1., instead of broadband, a dielectric resonator oscillator operating over a narow range of frequencies can be designed for narrowband permittivity detection as shown in Figure 4b [16].

## 2. Electrical Model of Cell in Suspension and Choice of Frequency for a Particular Application

**Figure 5:**
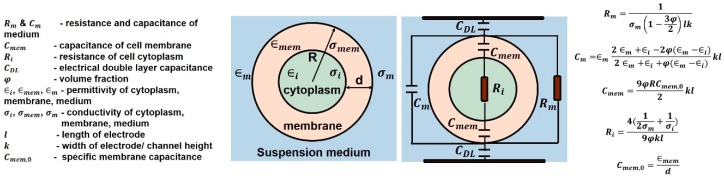
The equivalent circuit model of a single cell in suspension [17].

The electrical model of cell in suspension medium helps in explaining the variation of permittivity with frequency, in terms of capacitance and conductivity. Maxwell’s mixture theory [17] is used to derive the impedance of a single cell in suspension between two parallel electrodes as shown in Figure 5. The impedance of the medium is represented by resistor $R_{m}$ and capacitor $C_{m}$ in parallel. The membrane of a viable cell has very low conductivity and can be modeled as a capacitor $C_{mem}$, preventing current flow through the cell at low frequencies. The cell cytoplasm is represented by a resistor $R_{i}$, impeding current flow through it and the electrode-electrolyte interface is modeled as a capacitor $C_{DL}$, known as electrical double layer (EDL).

**Figure 6:**
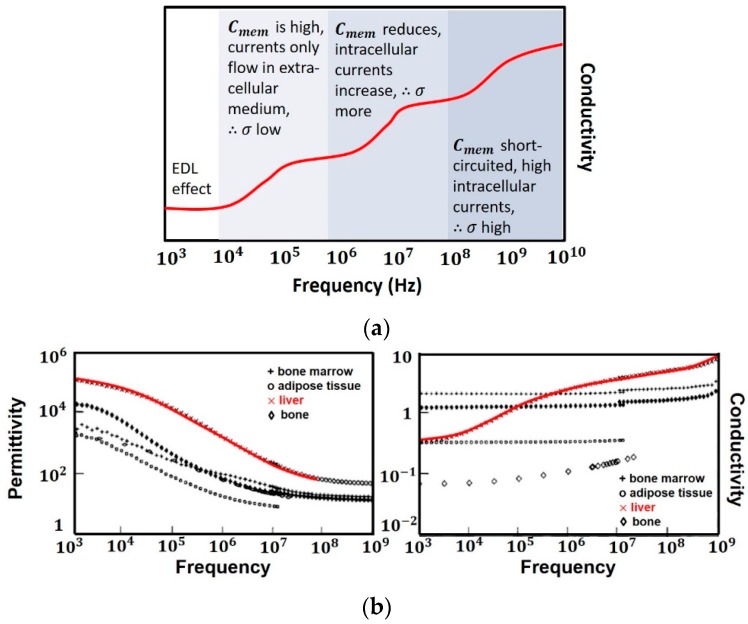
(**a**) Variation of conductivity of dielectric on application of electric field of increasing frequency. (**b**) Dielectric properties of liver of calf showing dispersion at ~1 MHz, reproduced from [18] by permission of IOP Publishing.

The presence of EDL, reduces detection sensitivity at very low frequencies (<1 kHz) as a majority of the voltage is dropped across EDL. Hence the impedance measurements in this range are of little use in sensing as they are dominated by EDL and are unable to provide information about cell properties (both inside and outside the cell membrane). The cell membrane has an approximate capacitance of 1 $\mu F/{\mathrm{cm}}^{2}$. At low frequencies (<1 MHz), $C_{mem}$ acts as an insulating layer and currents only flow in the extracellular medium, accounting for the low conductivity of tissues. Since currents cannot pass through the cell, intracellular information is still not accessible. When frequency is increased (1–100 MHz), the capacitive reactance $C_{mem}$ of cell reduces, increasing the intracellular currents and consequently conductivity. Further increasing the frequency (>100 MHz) makes $C_{mem}$ effectively short circuited, thus enabling the external field to pass through the cell cytoplasm. At these frequencies, the signal becomes sensitive to changes in the intracellular contents achieving maximum conductivity. Figure 6a shows the trend in conductivity on application of electric field to a dielectric and Figure 6b shows the dispersions in permittivity of a calf’s liver [18].

Thus, it is evident that at low frequencies (α and β-dispersion), information pertaining to cell membrane and extracellular fluid (cell shape, size, and membrane potential) can be obtained. This has been utilized in characterizing the different types of WBCs (e.g., lymphocytes, neutrophils and monocytes) [19] based on their different morphology and shape, thus different $C_{mem}$. At frequencies above which the applied field is able to penetrate the cell (γ-dispersion), intracellular information can be obtained. This range is useful for detection of biomarkers, glucose and DNA etc. (Table 1). Based on the application, the corresponding frequency band can be chosen. For instance, the dielectric response of live cells is fundamentally different from that of dead cells [20]. Live cells contain a large number of negatively charged molecules which attract positive charges (sodium and potassium ions) from suspension medium. The accumulation of mobile charges on the surface of the membrane gives rise to a membrane potential. In case of dead cells, pores are present, permitting exchange of ions with suspension medium. Thus, capacitive contrast of cell with respect to suspending medium is less compared to live cells [12]. Since this distinction is made on the basis of cell membrane potential, ∝- and β-dispersion regions can be utilized by subjecting the cells with frequencies of kHz–MHz range.

**Figure 7:**
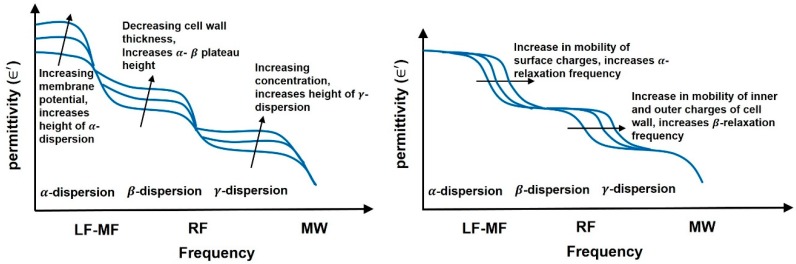
Variations observed in permittivity with change in membrane potential, membrane thickness, concentration and mobility of cells [20].

Figure 7 summarizes the effects on permittivity with varying cell thickness, cell potential, charge mobility and concentration of cells [20]. The plateau between ∝-dispersion and β-dispersion is affected by changes in membrane thickness, smaller the thickness, higher is the plateau. Larger the membrane potential, stronger is the ∝-dispersion, experimentally verified on muscle cells [22] where membrane potential controls the height of ∝-dispersion. The mobility of surface charges also affects the ∝-dispersion which moves towards higher frequencies with increase in mobility. Similarly, for β-dispersion, conductivities of outer and inner regions of cells control the length of β-plateau, while membrane thickness controls the height of β-plateau. Thus, by choosing frequency bands accordingly, biosensing applications can be performed, i.e., for observing changes in cell membrane, ∝- and β-dispersion regions can be utilized however, for applications where intercellular information is needed such as biomolecular detection, γ-dispersion region at microwave frequencies is used. Next we discuss the commercially available medical biosensors and their categorizations.

## 3. Commercial Medical Biosensors

**Figure 8:**
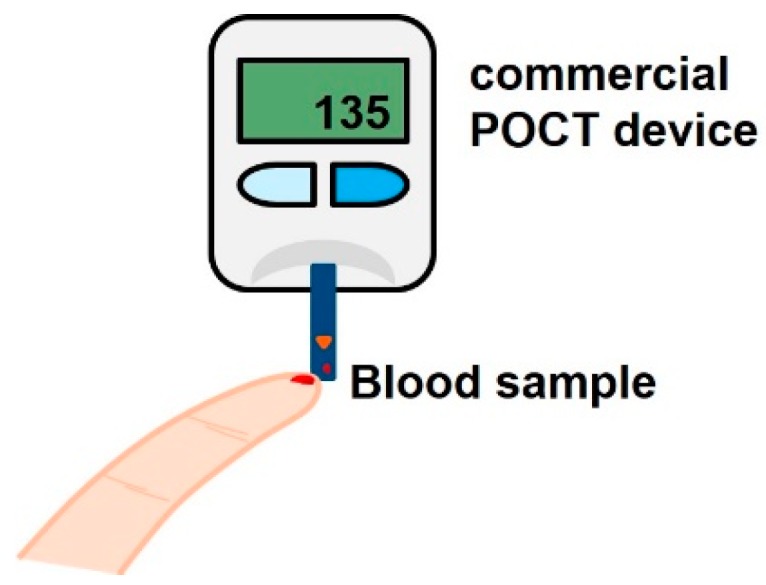
POCT device for home-based sensing such as diabetes, tuberculosis, HIV etc.

The first medical biosensor **,** for sensing oxygen during cardiovascular surgery, was reported in 1962 [23]. Since then there has been tremendous research in this field towards detection of cancer, glucose, genetic disorders and pathogens aiming for better reliability, sensitivity and selectivity while reducing time of acquisition of results. Point of care testing (POCT) [14] biosensors are becoming widely popular in the market due to their ability to perform a quick diagnostic test near the patient without the need for laboratory analysis (Figure 8). The commercially available sensors for glucose [24], HIV, tuberculosis, cholesterol, hCG (pregnancy), malaria and cancer are listed in Refs. [25,26]. These biosensors available on the market (Table 2) are based on electrochemical or optical methods. To the best of our knowledge, the only commercially available RF sensor is the vital sign sensor based on Doppler and a blood glucose monitoring device named Glucowise (MediWise, London, UK, Section 4.1.5). However, there is ongoing research in trying to develop microwave sensors with good repeatability and accuracy comparable with the state of the art commercial electrochemical and optical biosensors.

Notable efforts have been made in Ref. [27] in designing a non-invasive microwave blood glucose monitoring sensor using split ring resonators, where the in vivo and in vitro clinical interference test results were found to be comparable with those of invasive commercial glucose sensors. Another microwave glucose monitoring system, proposed in [28], based on split ring resonators was evaluated for its performance using clinical trials and shows promise in terms of integration into wearable devices.

RF biosensors for detection of biomolecules have undergone a large amount of research and have potential for commercialization as POCT devices. Detection of biomolecules require biorecognition elements [29], which recognize the target biomolecules and include antibodies, nucleic acid probes, bacteriophages and proteins. Antibodies can be monoclonal which recognize single epitope of target molecule, or polyclonal; capable of recognizing multiple epitopes of same target. However, they are expensive and require proper storage conditions to keep them from denaturing. Nucleic acid probes are attractive due to their associaition with genetic disorders, cancer etc. Single-strand DNA, hairpin DNA, peptide nucleic acid, DNAzmes and aptamers are commonly employed examples of nucleic acid probes. Phages are viruses that attack bacteria and use their replication system for phage duplication, hence can be employed for detection of clinically important bacteria.

Commercially available biosensors employ this biorecognition technique and transduce it to an electrochemical or colorimetric response. The home-based pregnancy strips measure the presence of human chorionic gonadotropin (hCG), a hormone secreted in urine during pregnancy by a strip pretreated with monoclonal antibodies, indicated by a red band in the test window of the strip. Other protein based sensors are for prostate cancer detection using prostate specific antigen (PSA) and for myocardial infarction using cardiac troponin I (cTnI). Glucometers are POCT devices for glucose and generally have colorimetric or electrochemical read-outs. In case of colorimeters, in presense of glucose, hydrogen peroxide or an intermediate product is generated to react with a dye and produce a color change proportional to glucose concentration. The popular enzymatic reactions being utilized in current electrochemical based glucometers are glucose oxidase, glucose dehydrogenase and hexokinase. POCT biosensors have also been developed for malaria, tetanus, C-reactive protein for meningitis, measles, mumps, influenza, AIDS, gonorrhea, filariasis and tuberculosis.

POCT has been realized as *lab-on-chip* [29] which is a miniatured device with capability to analyse several parameters. Using microfluidic channels and embedding antibodies, proteins or oligonucleotides allows multiple biochemical reactions to be analysed from a single drop of sample. Biosensors can be classified as labeled or label-free. In *labeled* techniques, the target by itself is not the transducer, but starts a series of events which then lead to a detectable signal. Enzyme-linked immunosorbent assay (ELISA) is one such example. The labels can be fluorophores, nanotags, quantum dots and secondary labeled antigens or DNA strands. Though labels offer high specificity, the main disadvantage of the labeling technique is the time required to label a sample. In *label-free* techniques, the target itself is the transducer and provides the detection signal. Surface and local plasmon resonances utilizing the evanescent field (Section 5.2.4) are examples of label-free methods. RF biosensors are label-free and minimally invasive, thus gaining popularity [14]. *Wearable POCT* devices continuously examine tear, perspiration and saliva for detection purposes such as blood alcohol content in drivers. *Wireless POCT* devices are also available and have been integrated into smartphones, for example, the Smartphone Optosensing Platform (SOP). In this the spectrum of the sample is observed from 450 to 600nm and a decrease in intensity indicates more neurotoxins, impying high susceptance to abdominal cramps, coma and hypotension.

Immunochromatographic Lateral flow assays (LFAs, also known as dipstick tests) and electrochemical sensors dominate POCT diagnostic market. Optical POCTs have an advantage over electrochemical method as the per-test cost is less. It obviates the need for electrodes and allows ease of multiplexing to detect multiple simultaneous reactions at once. *Passive integrated microwave* structures have potential in POCT diagnostics and is fabricated in standard semiconductor technology platform (CMOS or BiCMOS). In Ref. [30] an integrated passive device biosensor chip is designed for sensitive and reusable detection of glucose and uses interdigital capacitor (Section 4.2.4) in its resonator. Another example of glucose detection using passive integrated technology is given in Ref. [31] and uses rectangular meandered line resonator for sensing purposes.

**Figure 9:**
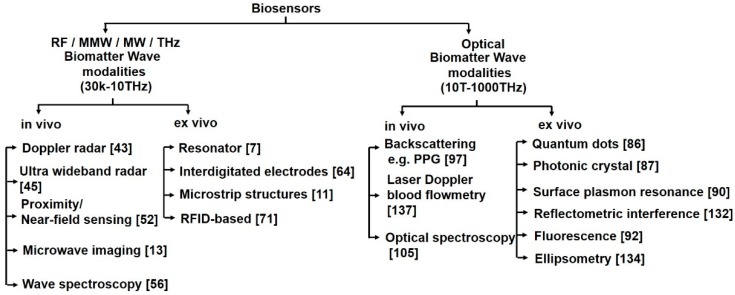
Classification of biosensors based on in vivo and ex vivo interaction modality of EM waves with biomatter.

In this paper, we have categorized EM wave sensors into labelled and label free, direct and indirect and in vivo and ex vivo sensors. In this paper, we refer to *direct* sensors as those sensors which provide intrinsic biomatter information by directly subjecting biomatter to EM waves and observe the unique permittivity signatures of biomatter (in case of RF/mmW/MW/THz sensors) and intrinsic optical properties e.g., amount of backscattered light (in case of optical biosensors). Proximity coupling based vital sign sensors (Section 4.2.5), photoplethesmography (Section 5.1.1), and CPW transmission line based sensors (Section 4.2.3) are examples of direct EM sensors. On the other hand, we refer to *indirect* sensors as those which measure the change in permittivity or optical properties. These are based on immobilization techniques which require bioreceptors to be fixed on the sensor’s surface and subsequently capture target analytes. This biorecognition event due to affinity binding between target and receptor, leads to a change in permittivity or optical property which can be sensed. Alternatively, indirect sensing using microfluidic channels can be utilized. Surface plasmon resonance (optical) and resonator based capacitive sensing (RF) are examples of indirect sensors. Thus, observing the intrinsic permittivity (e.g., when identifying cancer cells from normal cells) or changes in permittivity (e.g., when measuring concentration of analyte) forms the backbone of EM wave sensing. In case of optical biosensors, observing the intrinsic optical properties (e.g., for measurement of oxygen saturation using photoplethesmography) or observing changes in optical properties (e.g., for measuring the concentration of analyte through change in fluorescence, refection intensity, photoluminescence, interference pattern or refractive index) forms the basis of optical biosensors. These EM wave biosensors can also be categorized into in vivo and ex vivo biosensors. If the biosensors are able to sense without needing a sample to flow through them, i.e., they are able to detect abnormalities or vital signs in a living organism by simply keeping the sensor near the patient, they are known as in vivo biosensors. Alternatively, in ex vivo sensors the measurements are done in tissue/blood from an organism in an external environment with minimal alteration from environmental conditions. Our research group has developed a wearable CMOS dosimeter [32] and a self-optimizing IoT wireless video sensor node in Ref. [33]. However, in this paper we mostly focus on work from other research groups. The in vivo and ex vivo biomatter wave interaction modalities are listed in Figure 9.

## 4. Radio Frequency, Microwave, Millimeter Wave and Terahertz Biosensors

On application of an alternating field, with frequencies in the range of kilo to a few terahertz, the EM spectrum of biomatter shows several dispersions in permittivity, namely, α-dispersion in the kHz range due to ionic diffusion, β-dispersion in the MHz range due to interfacial relaxation and γ-dispersion in the GHz range due to dipolar relaxation. The choice of frequency is based on the information of interest (Section 2), e.g., changes in cell membrane potential are sensed in the α-dispersion and β-dispersion region, whereas, for obtaining intercellular information such as determing the concentration of biomolecules, γ-dispersion is used. Cell’s major constituent is water, thus change in concentration of biomolecule, will imply different water content and extent of dipolar polarization of water, which can be sensed. In biosensors based on affinity binding or microfluidic channels, the changes in permittivity due to concentration change are sensed *indirectly*. Whereas, the intrinsic permittivity of cells and tissues can be observed *directly* using CPW transmission line based sensors which provide a broad-band dielctric spectrum. When the entire dielectric spectrum is not required, resonators are employed and provide narrow-band dielectric information and permittivity is observed using network spectrum analyser in terms of relaxation frequency and s-parameters.

Some common applications of RF, mmW, MW and THz sensors include detection of cancer cells [34], glucose [35], DNA [13], fibroblast cells [36], other biomolecules [35] and vital signs [37] and are discussed in this section in detail. To understand the concept of affinity binding let us first discuss some common terminologies. (a) *Analyte* is a substance which is being measured or identified. (b) *Bioreceptor* is a biomolecule that recognizes the target analyte in sample solution. (c) The process of binding bioreceptors to the sensor’s surface is known as *immobilization*. (d) *Biomarkers* are measurable chemicals in an organism whose presense indicates a disease, infection or environmental exposure.

**Figure 10:**
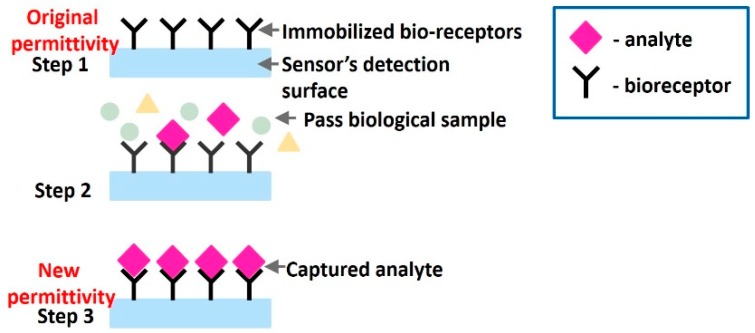
Biorecognition process on the surface of sensors. Bioreceptors are immobilized on sensor surface. On passage of analyte, interaction between target and bioreceptor causes permittivity change which is sensed.

Sensors extensively employ this bio-recognition process, where bioreceptors are immobilized on sensor surface, and the analyte to be detected is allowed to flow across the detection region to selectively interact with the already immobilized biomolecules causing change in permittivity (Figure 10). The sensors are designed to be extremely sensitive to changes in permittivity on its surface which is measured. Apart from immobilization, there is an alternative of using microfluidic channels, which are small channels allowing passage of analyte over the detection area of the sensor, sensitive to permittivity change.

In these sensors, generally capacitance sensing is employed. For example, a resonator is modelled as a capacitor and inductor in parallel, with change in permittivity, the capacitance changes. A resonator is a device that naturally oscillates at resonant frequencies. Thus, due to the change in capacitance, the resonant frequency changes, which is sensed. The different in vivo and ex vivo interaction modalities of biomatter with EM waves, namely RF, MW, mmW and THz are discussed next.

### 4.1. In Vivo EM Wave Interaction Modalities with Biomatter

As discussed earlier, in vivo sensing technique senses an abnormality or vital signs by placing the sensor near the living organism and exposing them to microwave radar without requiring samples to conduct measurements.

#### 4.1.1. Doppler Radar

In doppler based sensors for vital sign detection, such as heart rate and respiration rate, a continuous wave signal is emitted by a transmit antenna towards the subject’s time-varying chest wall. The motion of the chest wall is caused by cardiopulmonary action, which phase modulates the transmitted signal. The sensor then receives the signal reflected back from the subject via the receive antenna and demodulates it to yield the vital-sign information [38].

Several Doppler architectures have been proposed, such as direct conversion architecture, quadrature radar architecture, differential front end Doppler architecture and harmonic sensing architecture to name a few. Direct conversion Doppler radar [39] has free-running oscillators and is able to detect low-frequency cardiopulmonary signals without requiring phase-locked loops, however, has a null detection point leading to weak signals at every quarter wavelength from the sensor to the subject. This is overcome by using direct conversion quadrature radar architecture [40] where the upper and lower sideband frequency tones are generated by two voltage controlled oscillators and transmitted. Since it is difficult to avoid null detection problem for each sideband alone, hence, they are combined and the distance between the optimal and null detection point changes to a longer separation and avoided. However, it often introduces residual phase error, degrading detection accuracy. A differential front end Doppler radar is proposed in [41] which illuminates the body in two adjacent locations and are based on the fact that since only one of the beams illuminates the heart, the other can be used for motion cancellation. In [42] the subject is placed between two self-injection locking sensors for detection of heart rate using Doppler shift and the effects of random movements of the subject are cancelled by wireless mutual injection locking of the two sensors. [43] uses harmonic tags for subject identification and to isolate the thoracic body motion from the other limbs and thus enable Doppler radar to isolate subject’s respiratory motion from environmental clutter. A harmonic Doppler radar with heterodyne receiver is implemented for better SNR compared to homodyne architecture. Another advantage is the ability to work at a greater distance from the subject (more than 160 cm) while using the same transmitted power. In [44], the Doppler architecture transmits and receives signal via the VCO’s output and injection port respectively, and brings PLL to a self-injection locked condition. The frequency demodulator then extracts the vital sign information from the VCO output. This combination of injection locking and frequency demodulation achieves a high SNR and the null point detection problem is overcome by sweeping the VCO’s output frequency.

#### 4.1.2. Ultra Wide Band Radar

Apart from continuous wave radar, Ultra wideband radar (UWB) [45] can also be utilized for vital sign detection. It transmits repetitive short pulses in time and gets modified in its repetition frequency due to the body movements. In Ref. [46], ultra wide band microstrip antenna based breast imaging is done by illuminating the target with microwave signal and measuring the backscattered signal over a range of 3.92 GHz to 11.32 GHz in the near field of the antenna. It is imperative to keep the antenna close to the breast else the signal gets scattered from the breast skin. Discrimination of tumor cells from the healthy cells is done based on difference in dielectric properties of tumors and normal cells, i.e., microwaves are absorbed more by tumorous cells compared to normal cells. By noting the difference in conductivity (*σ*) and specific absorption rate (SAR), it is observed that specific absorption rate increases with increase in size of tumors. It is reported that @6 GHz, a healthy breast has *σ* = 0.7 A/$m^{2}$ and SAR = 2 mW/kg whereas a breast with tumor radius of 0.5 cm has *σ* = 1.2 A/$m^{2}$ and SAR = 5.2 mW/kg.

#### 4.1.3. Microwave Imaging

Another application of microwave radar in breast cancer imaging is seen in Ref. [13]. The system illuminates microwave signals to the target object and measures the backscattered reflection signals. γ-dispersion region is utilized to identify cancer cells from the normal tissue cells which vary widely in water content, and hence in complex permittivity. For instance, fatty tissue of healthy breast are transparent to microwave radiation compared to cancer cells containing more fluids due to lesions providing a higher lossy medium to the signal. Different antenna topologies have been established for improving the performance: [47] proposes a horn antenna operating in 1.5–6 GHz, UWB micro strip monopole antenna was proposed in [48] and a bow-tie antenna in [49].

#### 4.1.4. Proximity Sensors for Vital Sign Detection

**Figure 11:**
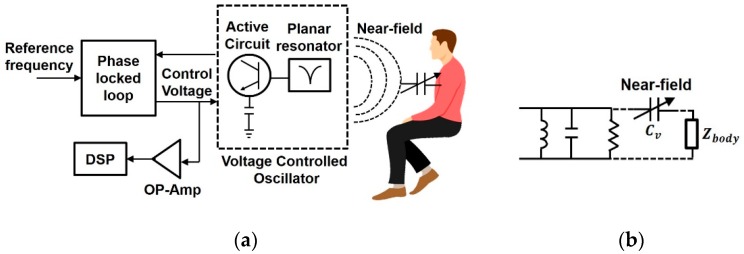
(**a**) Near field sensing using planar resonator. The resonator is part of the voltage controlled oscillator which is placed inside phase locked loop (PLL). The periodically moving chest is sensed through proximity coupling by the resonator and is transduced to a varying dc voltage in the PLL. (**b**) The time-varying chest movement is modelled as a varying capacitor $C_{v}$ and the resonator as a parallel RLC circuit.

The operating principle of proximity sensor is proposed in [50], where a subject is placed in front of a planar type circular resonator sensor operating at 2.4 GHz and is connected to a PLL (Figure 11a). On placing the target in the circular resonator’s proximity, the VCO’s oscillation frequency gets affected based on the dielectric properties of the human and its coupling to the resonator. The resonator can be modelled as a parallel RLC circuit and the human body movement due to cardiac activity as variable capacitance (Figure 11b). The net input impedance of the planar type circular resonator is a complex quantity and varies as a function of separation distance from the subject. Consequently, the oscillation frequency is also a function of separation distance from the subject and is converted to a varying DC voltage by the PLL (control voltage in Figure 11a). Frequency deviation is a critical factor and can be explained from [50,51] that for lower quality factors the deviation in oscillation is more which turns out to be beneficial for detecting vital signs, since the body movements are quite small to detect. An array resonator operating at 2.4 GHz placed in proximity of a wrist is used in [52,53] to obtain heart rate information. The reflection coefficient of the resonator/radiator varies as a function of distance between the resonator and walls of major arteries and this information is utilized to obtain heart rate information. Another proximity coupling RF sensor uses interdigital electrodes for planar RF resonator to transform the changes in blood volume to phase variations, proposed in [54]. The fringing electric fields existing among the fingers (Figure 11b) are distorted by the periodic pulse of the radial artery causing periodic changes in the oscillation frequency which is transformed to a variation in voltage by the PLL. A similar planar resonator operating at 2.4 GHz which acts as a near field radiator, senses vital sign information in [55] from the changes caused in oscillation frequency by the periodic movement of the body by respiration and utilizes a surface acoustic wave filter in detection circuit to enhance sensitivity.

#### 4.1.5. Wave Spectroscopy

As seen in Section 1, observing permittivity or alternatively scattering parameters for a range of frequencies can prove useful in medical applications. Choosing the frequencies in the microwave region allows detection of biomolecular concentration inside the cells. A similar approach is utilized in Ref. [56], where millimeter waves are chosen as shorter wavelengths permit use of a compact antenna while being large enough to penetrate the skin and reach blood profusion to obtain useful information. They develop a sensing device utilizing microstrip patch antennas operating at 60 GHz and measure the transmission coefficients to detect complex permittivity variations which is correlated to concentration of glucose in blood. This glucose monitoring device, known as Glucowise, is gaining wide popularity in the commercial market due to its non invasive feature which does not require blood samples as opposed to other state of the art optical and electrochemical biosensors.

### 4.2. Ex Vivo EM Wave Interaction Modalities with Biomatter

Ex vivo sensing techniques require a sample to flow across the detection surface to conduct measurements and obtain information. Affinity binding and microfluidic based sensors fall under this category.

#### 4.2.1. Resonator Based Capacitive Sensing Using Affinity Binding

Capacitive sensing is a common technique employed to measure the changes in permittivity. A resonator is a device which oscillates at resonant frequencies and can be modelled as a parallel RLC circuit. This modality is useful in measuring the concentration of biomolecules and for biomarker identification in a sample. Thus, bioreceptors are immobilized on the sensor surface to bind to the desired analytes (e.g., a cancer biomarker). The resonator before the affinity binding process has an intrinsic resonance frequency. When the analyte binds to the sensor surface, the permittivity/permeability in the near field region of the sensor’s surface changes, in turn changing the effective capacitance/inductance and consequently the resonant frequency, which is sensed.

**Figure 12:**
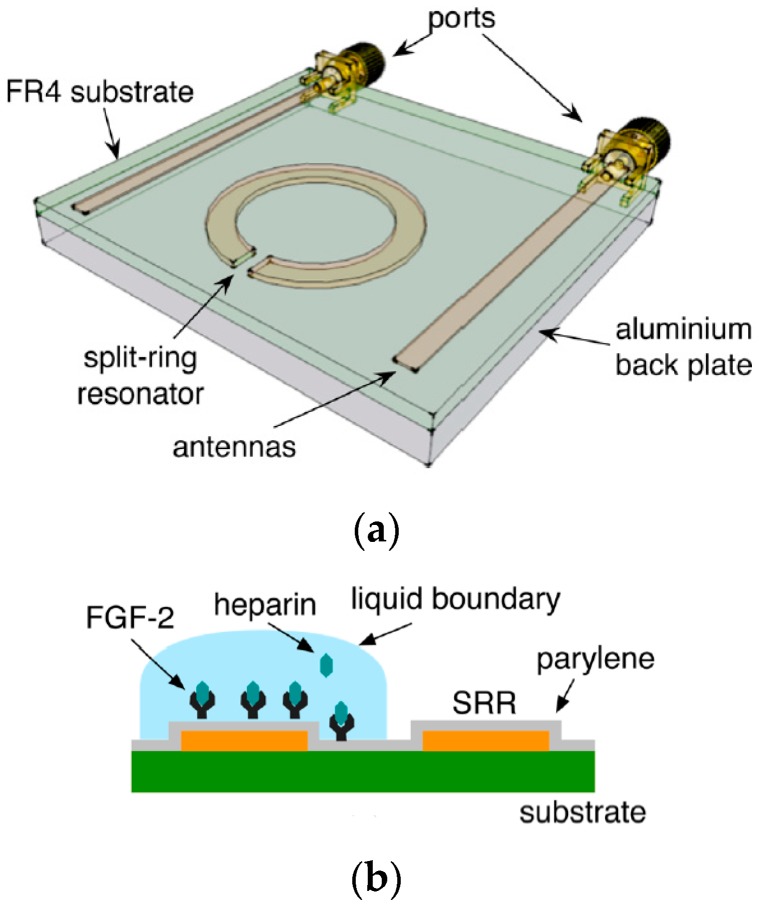
(**a**) Split ring resonator structure with integrated antennas on dielectric substrate. (**b**) Process of affinity binding of heparin to immobilized FGF-2 bioreceptor on split ring resonator surface. Reproduced from [7], with the permission of AIP Publishing.

Consider the example of a split ring resonator (Figure 12a) for sensing a biomolecule through affinity binding on its surface. The structure can be modeled using lumped elements to predict its resonance frequency as given in Ref. [7], then the resonance frequency $f_{m}$ is given by Equation (21):

(21) $$f_{m}=\frac{1}{2\pi \sqrt{C_{eff}L_{eff}}}\;$$

where $C_{eff}$ and $L_{eff}$ are the effective capacitance and inductance of the resonator respectively and are resonator geometry dependent:

(22) $$C_{eff}=C_{g}+C_{s},$$

**Figure 13:**
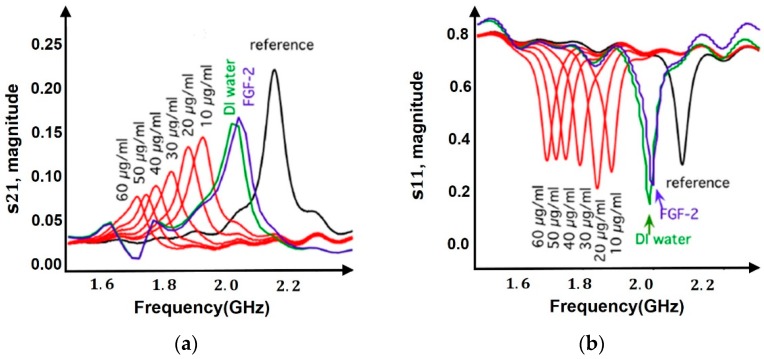
$S_{21}$ (**a**) and $S_{11}$ (**b**) parameter response of split ring resonator before and after affinity binding. The black curve is the intrinsic response of the resonance, on immobilization of FGF-2, the resonance shifts towards left (purple curve). On increasing the concentration of analyte (red curve) the resonance frequency shifts more and more towards lower frequencies, attributed to the increase in effective capacitance of resonator. Reproduced from Ref. [7], with the permission of AIP Publishing.

$C_{eff}$ can be decomposed into gap capacitance $C_{g}$, which models the capacitance associated with the slit of resonator along the current path and surface capacitance $C_{s}$, which is associated with the surface charges of the split ring. They are given as Equations (23) and (24):

(23) $$C_{g}=\varepsilon_{\mathrm{eff}}\frac{\mathrm{hw}}{g}+\varepsilon_{\mathrm{eff}}\left(h+g+w\right)$$

and:

(24) $$C_{s}=2\varepsilon_{\mathrm{eff}}\frac{\left(h+w\right)}{\pi }\ln\left(\frac{4r}{g}\right)$$

where *h*, *w*, *g* and *r* denote the height, width, slit gap and radius of the metallic split ring. $\varepsilon_{eff}$ denotes the effective permittivity of the media surrounding the metallic ring. Any changes in permittivity on the surface due to biomolecular binding is reflected in $C_{eff}$, which changes the resonance frequency and can be seen from scattering parameters through a Vector Network Analyzer as shown in Figure 13.

A split ring resonator (SRR) on being excited by EM waves through an antenna, EM wave’s emitted power gets coupled to SRR at its natural resonant frequency [7]. The frequency at which the emitted power couples to the SRR is seen as a drop in reflectance amplitude ($S_{11}$), as shown in Figure 13b and is known as its resonant frequency. On affinity binding of analyte to bioreceptor on the resonator surface, the permittivity changes which is transduced to a change in resonant frequency. This response of shift in resonant frequency with change in permittivity makes it a popular sensing device.

A planar split ring resonator is proposed for detection of prostate cancer biomarker [57] excited by a time-varying H-field component through a microstrip transmission line to induce surface currents on the rings. Anti-PSA and anti-cortisol were immobilized on the gold surface of the resonator by a protein-G mediated bioconjugation process and frequency shift of 30 MHz from intrinsic value was observed @100 ng/mL of PSA. A single element double SRR was demonstrated in [58] for DNA sensing where ss-DNA was immobilized on a Au surface. On coupling with complementary DNA a frequency shift of 60 MHz was observed on hybridization, from intrinsic value of 12.35 GHz. Similar approach was utilized in Ref. [59] where single-stranded DNA was immobilized on the split ring resonator. Subsequent binding of biotin followed by binding of streptavidin was observed as a drop in resonant frequency of 120 MHz and 40 MHz (@1 µM) respectively, from intrinsic value of 10.82 GHz. In Ref. [21], SRR excited by an antenna pair was used for detection of glucose and was immobilized by GOx enzyme. The measured resonant frequency shifts of 17.5 MHz in the $S_{21}$ parameters through a VNA gave the detected glucose concentration of 100 mg/mL and the shift increased in proportion to the glucose concentration. A similar split ring resonator topology, operating at 2.16 GHz was excited by EM waves and was used to detect change in concentration of heparin. A 10% shift in resonance was observed for a change in heparin concentration of 10 μg/mL [7]. Glucose detection using SRR was done in Ref. [21] using affinity binding by immobilizing GOx enzyme and the sensor’s biospecificity was confirmed by observing resonant frequency shift of 17.5 MHz on loading with glucose however, no effect was seen in resonant frequency on loading with fructose and sucrose.

In case of broadband biosensors, the required sample volume is of order of few milliliters. To reduce these volume requirements microfluidic structures integrated with coaxial lines have been demonstrated [60]. Resonator based biosensors have a sensitivity that is *Q* times higher than non-resonant structures (where *Q* is the quality factor), which implies lower loss. Whispering Gallery mode resonator has been employed in Ref. [61] where on incidence of EM waves, due to its circular geometry and low-loss characteristic, the wave undergoes several total internal reflections at resonator boundary and becomes confined inside the resonator, giving rise to resonances. An evanescent wave is present at the boundary of the resonator which is sensitive to changes in permittivity. This has been used for characterization of nanoliter liquids. Since different liquids have different water compositions, relaxation of water molecules in the liquid (which corresponds to a broad peak in dielectric loss between 5 GHz and 50 GHz) is used to determine the design frequency of the resonator, and 35 GHz is chosen. Changes in the inverse quality factor and shift in resonant frequency were used to map the permittivity of different liquids.

#### 4.2.2. Resonator Based Capacitive Sensing Using Microfluidic Channels

**Figure 14:**
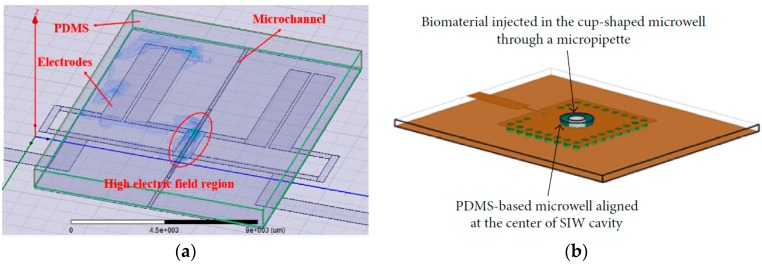
Examples of microchannels (**a**) Hairpin resonator having microchannels for passing analyte, reproduced from Ref. [8] with permission from Elsevier. (**b**) Substrate integrated waveguide (SIW) cavity resonator using microfluidic well [36].

Instead of resorting to immobilization and capture of analytes for detection, microfluidic channels through which analyte in suspension is passed over the detection area, can be used to detect the changes in permittivity as shown in Figure 14 [8,36].

The effect of change in permittivity on the s-parameters (reflection coefficient $S_{11}$)) can be related using Equation (25) for an ex-vivo sensor detecting glucose [16]:

(25) $$S_{11}=20\log\left|\frac{\left|Z_{in}\right|-Z_{0}}{\left|Z_{in}\right|+Z_{0}}\right|=f\left(Z_{in}\right)$$

where $Z_{0}$ is the characteristic impedance of the probe tip and $Z_{in}$ is the complex input impedance of the system (tube+air+glucose) and it can be estimated as Equation (26):

(26) $$Z_{in}≅jZ_{a}k_{a}\frac{\left(2t_{g}+t_{t}\right)-{k_{a}}^{2}t_{t}{t_{g}}^{2}\varepsilon_{g}}{1-{k_{a}}^{2}t_{g}\varepsilon_{t}\left(t_{g}+t_{t}\right)-{k_{a}}^{2}t_{t}t_{g}\varepsilon_{g}}=f\left(\varepsilon_{g}\right)$$

where $Z_{a}$ is the characteristic impedance of air, $k_{t}$ and $k_{a}$ are the wave vectors in silicon tube and air, $t_{t}$ is the wall thickness and $t_{g}$ is the diameter of cylindrical silicon tube. $\varepsilon_{g}$ and $\varepsilon_{t}$ are the dielectric permittivities of the glucose solution and silicon tube. $\varepsilon_{g}$ can be written as a function of concentration as Equation (27):

(27) $$\varepsilon_{g}\left(\omega \right)=\left[\epsilon^{\prime }\left(\omega \right)+c\dot{\delta }\right]-j\left[\epsilon^{\prime \prime }\left(\omega \right)+c\ddot{\delta }\right]=f\left(c\right)$$

where c is the concentration of glucose, δ = $\dot{\delta }-\ddot{\delta }$ is the increase in permittivity when the glucose concentration is raised by 1 unit, $\epsilon^{*}=\epsilon^{\prime }-j\epsilon^{\prime \prime }$ is the complex permittivity of water. Thus, increase in concentration *c*, increases $\varepsilon_{g}$ which maps to a change in reflection coefficient and resonant frequency.

Ref. [16] describes a microwave cavity resonator for measuring d-glucose concentration in blood. The impedance of the sensor is a function of concentration, and any change in concentration is observed as a shift in the reflection coefficient. d-Glucose is detected in the range of 150–550 mg/dL with a resonant frequency of 4.75 GHz, creating resonant frequency shifts of the order of MHz. A substrate integrated waveguide microfluidic cavity resonator was proposed in Ref. [36] for detection of fibroblast (FB) cells. Loading the microchannels with FB cells changes the effective permittivity leading to a change in effective capacitance which produces a considerable shift in return loss and resonance frequency. It showed that with increase in concentration of FB cells, the relative permittivity decreases causing an increase in resonant frequency from 13.46 GHz @0 FB cells/µL to 13.48 GHz @800 FB cells/µL.

A microfluidic based hairpin resonator operating at a frequency of 2.17 GHz is proposed in Ref. [8] for melanoma cell detection. It is a quasi-TEM mode resonator composed of two or more transmission lines of different characteristic impedances and the specific resonance frequency is determined by the length and characteristic impedance of the resonator. As mentioned previously, the resonance frequency changes with changes in dielectric properties in the coupling region, reflected as decrease in reflection coefficient ($S_{11}$) attenuation with increase in cell concentration.

#### 4.2.3. Microstrip Structure Based Capacitive Sensing-Coplanar Waveguide Transmission Lines, Open Stub & Shunt Stub

**Figure 15:**
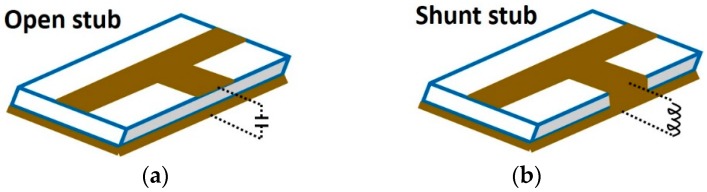
(**a**) Microstrip open stub. (**b**) Microstrip shunt stub microstrip structures used for sensing.

Microstrip structures include an open ended microstrip line, known as open stub as shown in Figure 15, modelled as having an input capacitance and conductance, and shunt-stub, modelled with input inductance and resistance. They are known to be sensitive to changes in permittivity on their surface and can be placed in an oscillator [62], such that the characteristic impedance and hence the frequency of oscillation is a function of permittivity. As a result, a material with unique permittivity $\epsilon^{\prime }$ and loss factor $\epsilon^{\prime \prime }$ will exhibit a unique oscillation frequency ($\omega =f\left(\epsilon^{\prime }\right))$ and output power $(P=f\left(\epsilon^{\prime \prime }\right)$). The proposed architectures show a frequency shift of 5% (27.8–26.4 GHz) for a change in permittivity of 2.4 (4.1–6.5) and have been used to identify different concentrations of methanol-ethanol. Ref. [63] uses an open stub as a near-field sensing element embedded in Colpitts oscillator. The open stub’s capacitance along with the oscillator’s oscillation frequency are a function of permittivity of the medium on top of the stub. Due to higher permittivtiy of calcium, it produces a greater shift in oscialltion frequency as compared to fat. It is made to operate at 28.7 GHz and is used to discrimate between fat and calcium.

**Figure 16:**
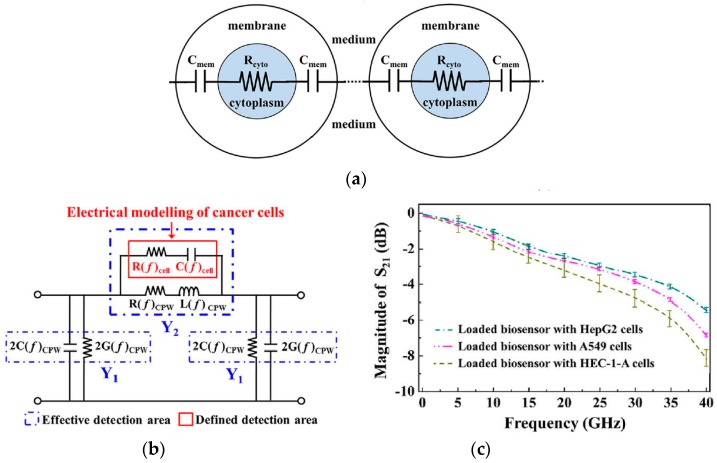
(**a**) Equivalent RC model of cancer cells. (**b**) Equivalent RLC model of CPW transmission line and cancer cells. (**c**) Broadband characterization of different cancer cells (HepG2, A549, HEC-1-A) over 1–40 GHz. Reproduced from [34] with permission from IEEE.

Capacitive sensing can also be done using coplanar waveguide (CPW) transmission lines. As mentioned before, dielectric characterization of cells in the microwave range provides a unique dielectric signature. A broadband characterization of human hepatoma (HepG2), human lung carcinoma (A549) and human endometrial adenocarcinoma (HEC-1-A) cancer cells over 1–40 GHz was demonstrated in [34] using a coplanar waveguide transmission line. Both CPW transmission line and cancer cells were modelled using RLGC circuit elements as shown in Figure 16 [34]. The equivalent resistance and capacitance of cell [34], were different for the different cancer cell types due to unique dielectric signatures which was sensed by observing the reflection coefficient over 40GHz bandwidth.

In [64] a DNA biosensor is proposed using coplanar waveguide as sensing surface. It was immobilized by AuNPs/MNPs (gold nanoparticles/magnetic nanoparticles), probe and capture DNA sequences to capture the target DNA (tDNA) and the shift in resonant frequency was observed with varying target DNA concentrations. A maximum observed shift of 0.9 GHz was obtained for a tDNA concentrations as low as 10 pM. Coplanar transmission line integrated with fluidic channel can be employed for cell quantification and counting as done in [65]. On passage of fluid through the channel the permittivity and the conductivity of the medium changes and corresponds to a unique dielectric signature and is shown as a plot of relative permitivity vs frequency over a range from 400 MHz to 35 GHz.

Another broadband approach using microwave coplanar waveguide (CPW) transmission line is employed in [11] for dielectric characterization of hepatoma G2 (HepG2) cancer cells over the range of 1–40 GHz. EM waves are able to penetrate the HepG2 cells at these frequencies causing gamma dispersion. Consequently, with a larger concentration of HepG2 cells, larger polarization effects are expected leading to a larger variation in complex permittivity. For sensing cell densities of 20 cells/μL to 2000 cells/μL attenuation constant and complex permittivity show distinct differences in cells over the range of 1-40 GHz. Another noteworthy mention is Ref. [66], which uses a similar principle of distributed microwave transmission lines for glucose concentration detection using a fluidic channel over 0-40 GHz and shows a resonant frequency shift of 0.54 GHz for a solution ranging from 0–347 mg/mL.

#### 4.2.4. Interdigitated Electrodes Based Capacitive Sensing

**Figure 17:**
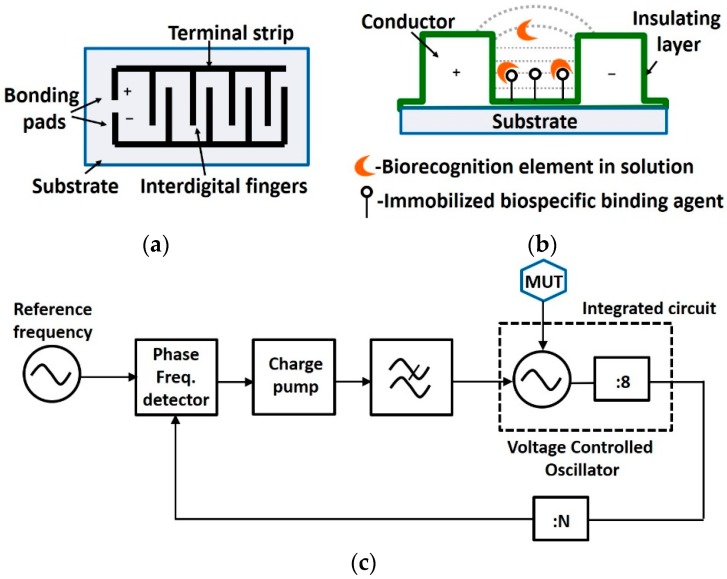
(**a**) Interdigitated capacitor (IDC) structure. (**b**) IDC sensing modality, bioreceptors are immobilized on the inner surface of IDT to capture target analyte. However, due to presense of fringing field, it is sensitive to bulk solution. (**c**) Interdigitated capacitor is embedded in oscillator circuit, such that the oscillation frequency is a function of permittivity of the material under test (MUT) passing through it. The oscillator is embedded in phase locked loop, whose voltage at the output of the charge pump is a function of permittivity.

An interdigitated capacitor (Figure 17a) is sensitive to changes in permittivity of the medium surrounding the detection surface (Figure 17b). On embedding it in an LC tank of a voltage controlled oscillator (VCO) (Figure 17c) [67] and placing that VCO in a PLL, a change in permittivity of the medium surrounding the interdigital capacitor, provides an opportunity to characterize materials e.g., methanol-ethanol. Change in permittivity causes change in capacitance and oscillation frequency. Thus, voltage output of charge pump in the PLL changes, which can be sensed. A high permittivity MUT increases the interdigital sensor’s capacitance, lowering the oscillation frequency of the VCO and increasing PLL’s tuning voltage. A similar topology in [68] is shown to have detected sample volume of 10–20 µL in 7–9 GHz range.

Another permittivity sensitive PLL is proposed in [67] over the frequency range of 19.2 GHz to 20.8 GHz for characterization of medically relevant liquids. A permittivity controlled interdigital capacitance sensor is embedded in an oscillator whose capacitance gets modulated with the inflow and outflow of cells in biological suspension on top of the sensor. These capacitive pulses are defined by the concentration and velocity of the particles and result in frequency modulation of the oscillator. A PLL demodulator enables a dynamic detection approach giving a pseudo DC (few kHz) signal and for particles with different dielectric permittivity it translates to different height of voltage pulses. In [69] the dynamic capacitive sensor is shown to be capable of detecting frequency changes of 100 PPM, corresponding to a shift of 1.43 MHz from 14.3 GHz operating frequency.

Interdigital capacitance sensors along with a meandered inductor are set in parallel and implemented in a microwave coplanar waveguide for distinguishing colorectal tumor cells from cell permittivity measurements in [70] and reported a frequency shift of 112 MHz with an operating frequency of 12.5 GHz with just three SW620 cells (found in third cancer stage).

#### 4.2.5. Radio-Frequency Indentification (RFID)-Based Biosensor

An interesting approach is proposed in Ref. [71] operating at 915 MHz, where the reflected RF signal strength varies with change in concentration of target analyte. The resulting signal, for high target concentration is strongly reflected compared to that of a low target concentration.

**Figure 18:**
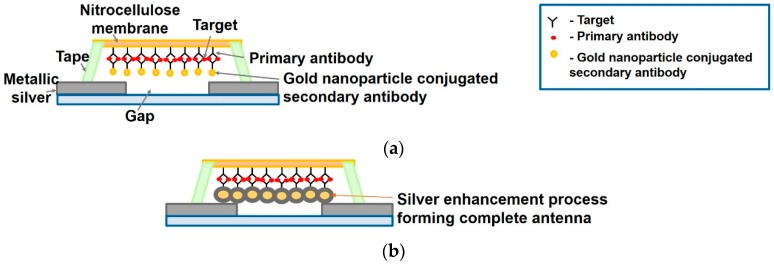
(**a**) Antibodies (anti-IgG) are immobilized on nitrocellulose membrane and bind to target (IgG). (**b**) Silver enhancement process is done on the complex antigen-antibody sandwich which self assembles a chain of micromonopole antennas. As the size of the silver-enhanced particles grow, the chain of microantenna segments bridges the gap between the split antenna which reflect the impinging RF signals at a desired frequency [71].

For this, an existing dipole antenna is split and covered with a nitrocellulose (NC) membrane. Target specific antibodies (anti-IgG) are immobilized on the NC membrane to capture the analyte (IgG). This is followed by a silver enhancement process on the complex sandwich structure formed by antigen-antibody hybridization as shown in Figure 18. Silver enhancement process self-assembles a chain of micromonopole antennas. As the size of the silver-enhanced particles grow, the gap between the split dipole structure is bridged, forming a complete dipole structure.

On impinging RF waves, it reflects at a desired frequency, but varies in strength based on target concentration. The strength of the relected signal or alternatively the detection distance is sensed using an RFID reader. For a 2 μL increase in IgG [71], the normalized detection length of the antenna increases by 30%.

### 4.3. Terahertz Biosensors

Terahertz biosensors are gaining a lot of popularity and deserve a special mention. The frequency range of 0.1 THz to 10 THz [72] is of relevance as it coincides with the vibrational frequency of some importamt biomolecules, thus changes in the concentration of biomolecules can be viewed as a shift in resonant frequency in the dielectric spectrum. The advantages of these THz biosensors over other EM wave sensors is their improved sensitivity, detection limit, volume of sample and cost.

The terahertz frequency band is in accord with the vibrational and rotational energy levels of important biomolecules, for instance, cancer biomarkers and thus its spectrum contains abundant information about their configuration and conformation. Studying the protein-protein interactions is vital to gain insight into several natural processes occurring inside every living being. When the different molecules interact, their molecular conformation changes, changing the location as well as the intensity of the characteristic resonant peaks in the spectrum, thus enabling it to be probed [73]. This technique is promising as it offers label-free and non-contact inspection of biomolecules.

Terahertz biosensors employ similar techniques as RF, MW and mmW biosensors. The following examples utilize split ring and microfluidic based resonators also employed by RF, MW and mmW sensors. In [73] hybridized and denatured DNA was distinguished by comparing the difference in resonant frequency shift in ssDNA. Another work [74], proposes the use of metamaterial based THz sensor due their their high sensitivity for detection of biomolecules. Rat IgG’s concentration was detected by observing frequency shifts in resonant peaks. Split ring resonators integrated with microfluidics was designed in [72] from 0.1–1 THz for detection of liver cancer biomarkers in early stage, namely Alpha fetoprotein (AFP) and gGlutamine transferase isozymes II (GGT-II). In [75], THz spectroscopic analysis of genetic sequences is demonstrated and a distinct refractive index is observed for each binding state of the DNA sequences.

### 4.4. Discussions

Microwave biosensors have become popular due to several advantages they offer. The most important one being non-invasive and lable free, unlike some optical biosensors which employ fluorescence and quantum dot labelling for detection. The label free optical methods include surface plasmon resonance and interferometer based biosensors. For instance, to distinguish between living and dead cells, staining or fluorescent markers employed may interfere with the cell. Instead, the difference in dielectric properties of living and dead cells, attributed to presence of pores in dead cells allowing passage of chemicals through the membrane and providing a greater capacitive contrast in living cells as compared to dead cells, can be employed for sensing using microwave frequencies. Microwave sensors also provide high sensitivity, selectivity and real time monitoring of biological reactions [68,76].

Immobilization is one of the methods employed for ex vivo detection, where the bio-receptor is fixed on the sensor surface to trap target analytes leading to a change in permittivity of the medium surrounding the sensor. The other option is to incorporate microfluidic channels on the sensor surface through which the analyte in suspension flows and a change in permittivity can be sensed. A drawback of capacitive biosensors employing immobilization of biorecognition layer is that if the layer is not insulated properly, it leads to ions flowing through the layer, short circuiting the sensor system [77]. On the other hand, using microfluidic channels allows precise automated fluid delivery with reduced reagent consumption flowing in an enclosed low cost system [78]. Capacitive sensing employed in microwave and RF biosensors may utilize components like interdigitated electrodes, resonators (such as split ring or cavity or whispering gallery mode) and microstrip line structures (such as CPW transmission line, open and shunt stub). These are modelled as RLC circuits with the capacitance being a function of permittivity. Resonators, interdigitated electrodes and microstrip structures are generally part of an oscillator circuit, whose resonant frequency changes with changes in capacitance. Thus changes in biomolecular concentration is traced to changes in capacitance or resonant frequency shifts. Resonators are employed for narrowband detection of permittivity, observing changes over a small range of frequencies, while broadband detection is made possible using CPW transmission line-based sensors. The merits and demerits of employing these ex vivo techniques are discussed next.

Resonator-based circuits enable sensitive differentiation of dielectric properties of material being tested and is seen as a difference in magnitude and frequency shift of s-parameters at resonance frequencies (Figure 13). They are advantageous in terms of small size, fast response and real-time measurements. The disadvantage of resonant based methods is that data can only be obtained at discrete frequencies, whereas in broadband; continuous permittivity spectrum is obtained. Coaxial resonators proposed in [79] are comparatively less bulky, more robust and easier to integrate with microfluidics than cavity and hairpin resonators. However, the dimentions of resonator determines the resonant frequency, hence tradeoff for miniaturization. The potential for miniaturization is limited in coaxial resonators, as further reducing the diameter of the coaxial cable will significantly reduce the quality factor, reducing the resolution. Thus, in cases such as detecting a non-polar substance with no significant frequency dispersion, the extra information provided by a coaxial resonator may not be required and instead we make use of much simpler and smaller split ring resonator. However, planar split ring resonators suffer from high conduction losses [78] as the thickness of resonator is comparable to skin depth at low GHz frequencies.

Capacitive sensing using interdigitated electrodes has its limitations due to its sensitivity to changes in bulk solution, as shown in Figure 17b. This is due to the fact that the electric fields curve out into the solution, on encountering a change in permittivity, thus the effective capacitance of interdigitated electrodes is affected due to near field sensing effect [11,66,77].

CPW transmission lines have shown to provide wide bandwidth dielectric characterization of cells using frequency dependent parameters, i.e., complex permittivity. However, the measured curves of medium and cells are similar [8] making it difficult to distinguish between them unless there is high dielectric permittivity contrast between the cells and surrounding medium.

Coming to the in vivo sensing methodologies, doppler has received attention as a remote monitoring system for applications such as cardiopulmonary monitoring for detection of sleep apnea and other vital activities. One of the major challenges of Doppler is that the vital sign information gets buried by signals due to body movement and researchers have tried to curb these motion artifacts [80]. Ultra wideband (UWB) detection is considered better than Doppler as it has no null point detection problem. However, UWB detection requires a software controlled delay line for control gating. Also, information about the distance between the radar and the subject needs to be provided to the microcontroller to program the correct delay. In UWB radar, utilizing short wavelengths of Ka band (26.5–40 GHz) has several advantages. It is more sensitive to small displacements, i.e., a shorter wavelength generates a larger phase modulation, requiring a smaller antenna and has improved signal to noise ratio [45]. Proximity coupling is a better alternative to Doppler, due to the fact that Doppler sensors basically transmit and receive signals, hence not adequate for multiple subjects in their detection range. Microwave signals are suitable for sensors with motion detection capability such as Doppler, in which phase displacement between the transmitted and reflected signals can be mapped to physical movement of reflecting surfaces. Microwave frequencies are suitable for this purpose as the physical displacements of arteries and organs are comparable to microwave wavelengths which are of the order of cm in air but mm in tissues, thus suitable for phase detection. A list of some common RF, MW, mmW and THz biosensors are given in Table 3, Table 4, Table 5 and Table 6. 

## 5. Optical Biosensors

The interaction of biomatter with EM waves in the range of 300 G–790 THz is marked by distortion polarization seen as two distinct resonances in the permittivity spectrum (Figure 3). Direct and indirect biosensing techniques are commonly employed in both microwave and optical biosensors. Direct optical sensors, such as photoplethesmograms and laser doppler flowmeters are based on observing the backscattered light from arteries. Indirect optical sensors employ affinity binding or microfluidic techniques and observe change in permittivity in terms of optical properties, such as reflection spectrum, photoluminescence, refractive index, surface enhanced raman spectrum and fluorescence.

Similar to the case of RF/mmW/MW/THz biosensors, here too, affinity binding of immobilized bio-receptors on sensor surface is used to capture the target analyte, which changes permittivity on occurance of a biorecognition event. The sensors are designed to be extremely sensitive to changes in permittivity on its surface which is measured. Apart from using immobilization techniques, microfluidic based optical detection is becoming popular in POCT diagnostics. These include applying conventional optical detection methods such as absorbance, fluorescence, interferometry and surface plasmon resonance in microfluidic biosensors [84].

Optical sensors [85] can also be classified into in vivo and ex vivo as discussed earlier and based on this categorization different interaction modalities have been classified in Figure 9. Optical biosensors are popularly used in the medical domain for detetction of cancer biomarkers [86], biotin [87], avain influenza [88], immunoglobulins [89], DNA, olegonucleotides, hormones [90] and vital sign detection [91]. The commonly employed modalities are interferometry [88], fluorescence spectrometry [92] and surface plasmon resonance [93] and are discussed next.

### 5.1. In Vivo EM-Wave Interaction Modalities with Biomatter

Visible light biosensors operate in the frequency range of 430 T–790 THz, or in terms of wavelength, from 400 to 700 nm. Whereas, infrared (IR) biosensors operate in the frequency range corresponding to 300 G–430 THz and wavelengths corresponding to 400–700 nm. They are very popular due to the advantages offered [94] such as sensing speed, immunity of signal to electromagnetic interference (EMI) and ability to obtain high information content. The following are some common in vivo visible light and IR interaction modalities with biomatter.

#### 5.1.1. Backscattering for Applications Like Photoplethysmography

**Figure 19:**
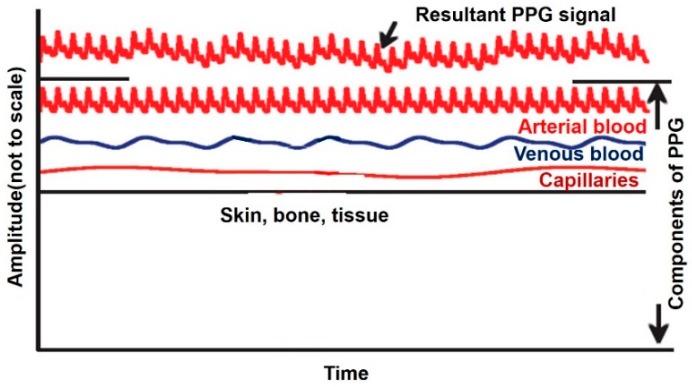
Components of PPG signal: the a.c. component due to arterial blood flow and the d.c. components by other tissues, bone and skin. Reproduced from Ref. [91] with permission from IEEE.

Blood is known to absorb more light than surrounding tissue, hence a photoplethysmography (PPG) senses the amount of backscattered light to derive information. For, instance a reduction in amount of blood leads to an increased intensity reflected light [95]. PPG waveform comprises two components, the d.c. component is a result of reflected signal from tissue and an a.c. component corresponding to change in blood volume during systole and diastole [96] (Figure 19 [91]). There are two possible configurations for PPG, namely, transmission mode, where the light source and sensor are not on the same side of the tissue and reflection mode where both light source and sensor could be on same side [97].

PPG has been extensively used for pulse oximetry which operates by emitting two light wavelengths, red and infrared through the body, generating two PPG signals. Red light is markedly absorbed by deoxyhemoglobin (Hb) and the infrared by oxyhemoglobin (Hb$O_{2}$). A ratio of the Hb PPG signal to Hb$O_{2}$ siganl is used for acquisition of pulse oximeter oxygen saturation (Sp$O_{2}$).

Portable and wearable PPG sensors are prone to motion artifacts [91] from body movements. One method of overcoming this issue is demonstrated in Ref. [91] by designing a two-stage normalized least mean square adaptive noise canceler using real-time adaptive algorithm. Another approach for motion artifact cancellation is proposed in Ref. [98], where motion artifact’s frequency response is observed to be in the same frequency band as PPG signals. By shifting the PPG band away from motion artifact band using an a.c. source instead of d.c., resolves the issue.

PPG signals indicate respiratory induced intensity variations documented in Ref [99]. Apart from heart rate and blood oxygen saturation level measurements, PPG is also commonly utilized for determining the cardiac output which can be estimated from the PPG derived pulse signal (cardiac output=product of stroke volume and heart rate). PPGs have been proposed for peripheral arterial occlusive disease detection, assessment of endothelial function and detection of vasospastic condition [100]. The second derivative wave of PPG signal is called acceleration PPG (APG) and is an indicator of acceleration of blood. APG siganls can be used to detect blood pressure, risk of coronary heart disease [101], migrane [102] and presence of atherosclerotic disorders [103].

#### 5.1.2. Laser Doppler Blood Flowmetry

A beam of light is made to fall on human tissue and the moving blood cells traversing through this volume, reflect light in a Doppler shifted manner whereas the surrounding tissues reflect it in an unshifted manner. This shifted light’s magnitude and frequency can be used to map the number of moving cells and their velocity. This technique is known as Doppler blood flowmeter and has been used in Ref. [104] where they measure blood flow in the earlobe.

#### 5.1.3. Optical Spectroscopy

Like microwave spectroscopy, near infrared spectroscopy (NIRS) can also be utilized for medical applications. One proposal is to measure cerebral blood flow and concentration of oxy- and deoxy- hemoglobin to gain insight about neural behavior as concentration of glucose and oxygen increases during neual activity. A technology named high density diffuse optical spectroscopy (HD DOT) is undergoing a lot of research by Openwater. DOT incorcopated with NIRS is based on transmitting ultrashort pulses of IR radiation and detecting varying diffused light a few centimeters away from the brain. Comparing the predicted arrival times with the actual diffused light arrival times, hemoglobin concentration is detected [105]. It is under deliberation if this promising technology will allow telepathy and enable mind reading.

### 5.2. Ex Vivo EM-Wave Interaction Modalities with Biomatter

The following are some common ex vivo visible light and IR interaction modalities with biomatter.

#### 5.2.1. Quantum Dots

**Figure 20:**
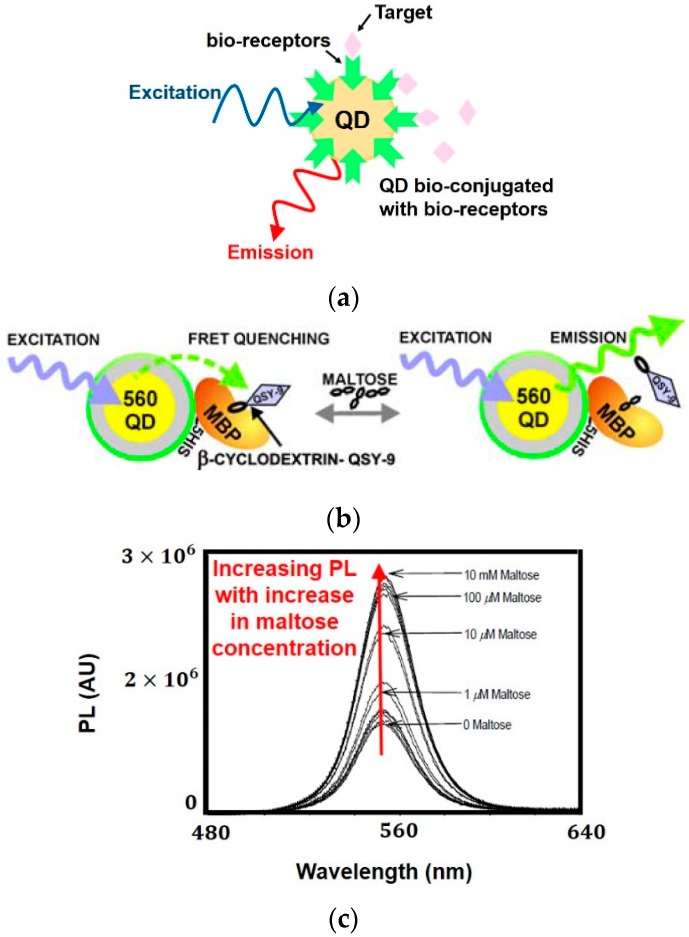
(**a**) The interaction modality of bioreceptor immobilized QD with target, on application of visible light leading to emission of certain wavelength dependent on the physical properties of QD [86]. (**b**) QD sensing maltose concentration. (**c**) Effect on photoluminescence of QD with increase in maltose concentration [86].

Quantum dots (QDs) are semiconductor nanocrystals which emit light of specific frequencies depending on the dot size, shape and material, on being excited by EM waves. For sensing purposes, QDs are immobilized with a bioreceptor to capture target analyte (Figure 20a). This biorecognition process modifies the dot’s electric field effects (due to change in structure by attachment of target biomolecules) leading to a change in photoluminescence (PL) which is sensed [86,106] as shown in Figure 20b,c. The sensitivity of QDs to external field has been used for neuronal cell action potential imaging [107] to help understand the cellular signaling pathways involved. QD’s have been used as a probe in [108,109] for detecting bovine serum albumin and nucleic acids where DNA binding leads to a linear increase in photoluminescence. Another application is to stain B-cell/T-cell antigens with different colored QDs in fixed lymph nodes [110].

They are also employed in immunoassay detection of cancer biomarker. These include, QD staining of CA125 tumor marker for monitoring ovarian carcinoma [111] and for monitoring proliferation of peroxisomal membrane protein in liver cells [112]. QD-immunolabeling by conjugating to primary and secondary antibodies is effective in detection of bacterial cells and protozoan cells [113,114].

#### 5.2.2. Photonic Crystals (PCs)

**Figure 21:**
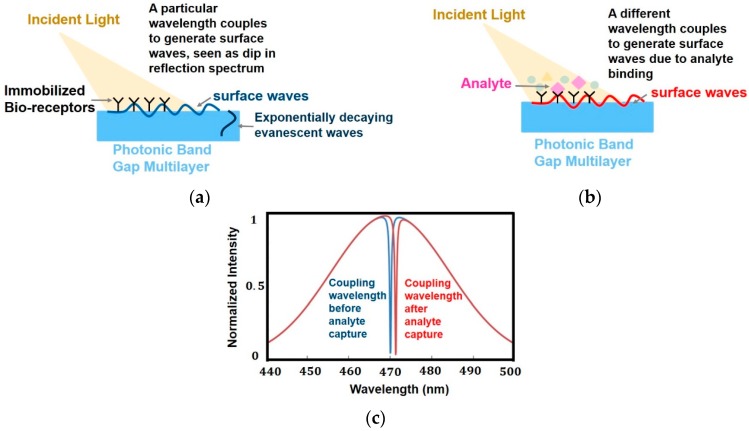
(**a**) Sensing modality using photonic crystal. On being excited, a particular wavelength couples to the photonic crystal leading to formation of surface waves, seen as a dip in reflection spectrum. (**b**) After capturing the analyte, the permittivity changes and a different wavelength couples to form surface waves. (**c**) Change in coupling wavelength of photonic crystal before and after analyte capture [87].

PCs are periodic dielectric materials made of alternating refractive index regions. Multiple reflections from surfaces may destructively interfere, forming forbidden band gaps, preventing propagation of certain wavelengths. When light is incident on a photonic crystal with photonic band gaps, a certain wavelength couples to it and gives rise to surface electromagnetic waves (Figure 21a). This is indicated by a sharp drop in reflection spectrum at that particular wavelength as shown in Figure 21c. These electromagnetic surface waves are called photonic crystal surface waves (PC SWs) and travel along the interface of different dielectric constants (e.g., photonic crystal and air) and decays vertically from the interface, these EM waves are known as evanescent waves. The sensing action is based on the fact that evanescent waves are very sensitive to mass loading or change in refractive index. On affinity binding of bioreceptor to target analyte at the interface (i.e., the photonic band gap film aund air), alters the surface wave coupling conditions (Figure 21b) and a different wavelength of light couples to the photonic band gap film forming surface waves. This can be seen from a shift in dip in the reflection spectrum.

This has been effectively used in characterization of liquids with different refractive indexes such as methanol/ethanol and for detection of bovine serum albumin (BSA) [87] through affinity binding by immobilizing the photonic band gap film with an antibody.

A biosensor based on PC SWs can be used such that s-polarization contains information about the PC SW excitation angle and is sensitive to adlayer (absorbed layer) thickness and refractive index (RI) of analyte suspension, while p-polarization is used to contain information about the critical angle of total internal reflection which is sensitive to RI of the liquid only. Combining these two allows registration of adlayer thickness and RI of liquid. This has been used in detection of free biotin which binds to immobilized streptavidin on PC surface [115].

#### 5.2.3. Interferometry

**Figure 22:**
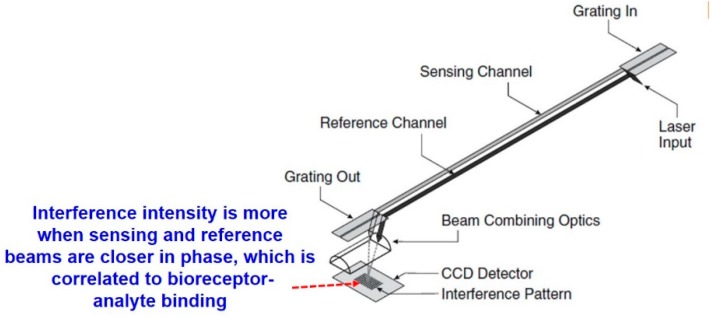
Interferometer combining sensing and reference beams to form an interference pattern used for detecting permittivity change. Reproduced from Ref. [88] with permission from Springer Nature.

As explained before, evanescent electromagnetic field is very sensitive to changes in refractive index. Thus, coupling a bioreceptor (such as an antibody) to the waveguide surface, binds to the analyte (such as antigen) and alters the original refractive index. This can be detected using an optical interferometer where a reference beam is optically combined with the sensing beam creating an interference pattern as shown in Figure 22. The phase difference between the reference and sensing beams can be corelated to bioreceptor-analyte binding on PC surface. For instance, if there is not much target in the sample solution (negligible target binding), the reflected and sensing beams will almost be in phase and the interference intensity will be strongest. Detection of avian influenze virus is done in Ref. [88] using this technique by immobilizing polyclonal antibody on PC surface. Refractive index changes of less than 10^−6^ can be detected using this technique with a sensitivity of 0.01 rad.

#### 5.2.4. Surface Plasmon Resonance

**Figure 23:**
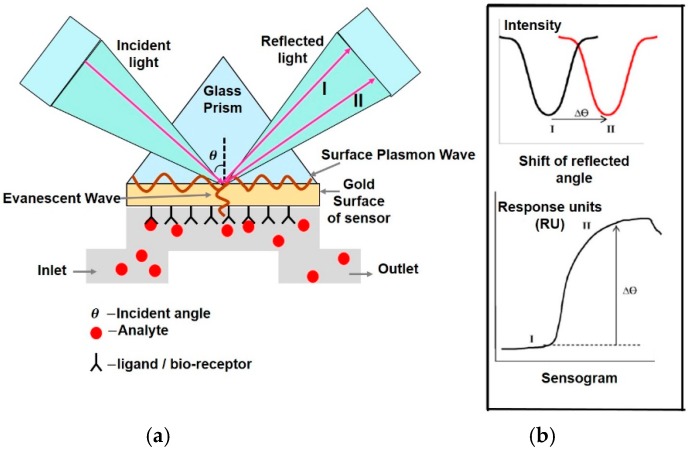
(**a**) SPR phenomenon–incident light is directed on a glass prism coated with thin gold layer. A part of the reflected light is transformed to evanescent wave which transfers energy to surface plasmons. At certain angle θ the momentum of the incident light matches the momentum of the surface plasmons and SPR occurs, seen as a dip in intensity of reflected light at a specific angle of reflection. (**b**) The reflected angle changes from I to II on reaction of analyte with ligand. The changes in reflected angle are plotted in sensogram in RU [116].

Surface plasmon resonance (SPR) has a similar principle as photonic crystal surface waves. Surface plasmons are coherent delocalized electron oscillations existing at interface of two materials. When light is incident on the sensor surface, a part of the reflected light is transformed to evanescent waves which transfers energy to surface plasmons as shown in Figure 23a [116]. 

When the momentum of the incident light matches that of surface plasmons, it gives rise to SPR. This is observed as a dip in intensity of reflected light at a certain reflected angle (Figure 23b) and is very sensitive to changes in the refractive index of the medium.

**Figure 24:**
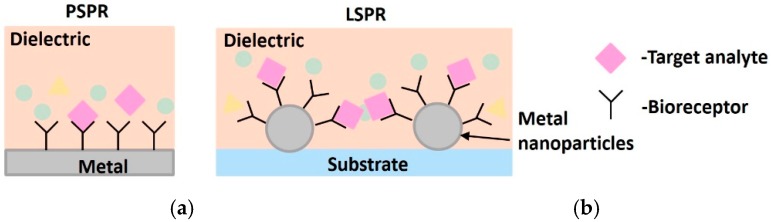
(**a**) Propagating SPR (PSPR) propagates along the metal dilectric interface. (**b**) Local SPR (LSPR) is non-propagating and occurs on metallic nanoparticles.

Thus SPR sensors are optical refractometers capable of sensing changes in the dielectric medium at the interface during affinity binding of target analyte and bioreceptors. It modifies the characteristics of the incident beam such as angle, wavelength and phase which can be detected. The angle at which light is reflected from the sensing surface is known as SPR angle and the response signal is expressed in RU (response units) which is directly proportional to mass loading. These can be categorized into propagating SPR (PSPR) and Local SPR (LSPR). PSPR is excited on continuous metal films and propagates along the metal/dielectric surface upto several μm whereas LSPR is excited on metallic nanoparticles and is non-propagating as shown in Figure 24.

**Figure 25:**
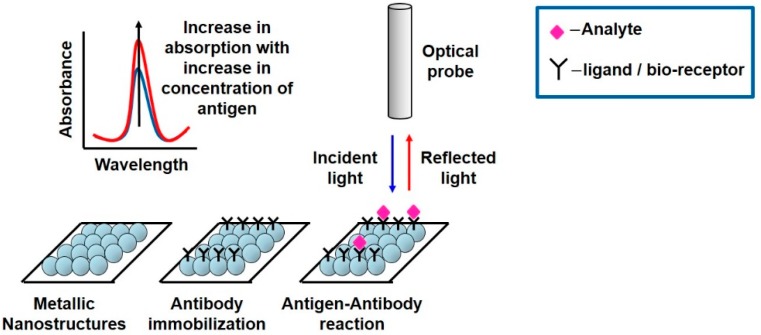
Local surface plasmon resonance phenomenon occurring due to surface plasmons of metallic nanostructures. The wavelength which is responsible for this phenomenon is seen as a peak in absorption spectrum.

Nanoparticles such as gold and silver are known to have strong absorption in the visible spectrum. Local surface plasmon resonance occurs when the incident photon frequency matches the collective oscillations of conduction electrons of metal nanoparticles. The wavelength which causes LSPR can be seen as a peak in the absorbance spectra as shown in Figure 25. Antibodies are immobilized on the nanoparticles and the concentration of antigens is detected, through antigen-antibody interactions which changes the peak absorption intensity of LSPR spectra. With a larger concentration of antigens binding to the antibodies, more nanoparticles optically couple to the incident light and result in more absorption of energy at that wavelength. The sensing capability of these nanoparticles can be modified by changing their size, shape and material.

Detection of human epididymis secretory protein-4 (HE4) biomarker is proposed in Ref. [117] for early diagnosis of ovarian cancer using a localized surface plasmon resonance biosensor. Anti-HE4 antibody probe/bioreceptor is assembled on sensor surface using amine coupling and a shift in the extinction maximum of LSPR spectrum is used for sensing.

A high resolution LSPR biosensor is proposed in [118] with a density resolution of 35 fg × mm^2^ which corresponds to less than one DNA molecule per nanoparticle. A narrow beam of light is incident on the base of the prism and undergoes total internal reflection which excites the LSPs via evanescent field. The amplitude and phase of the reflected light get modulated based on polarization of incident light and refractive index in the vicinity of metallic nanoparticles. With the incident light being linearly polarized, the reflected light comes out to be generally elliptically polarized. The parameters of this elliptically polarized light vary with the RI of the vicinity of nanoparticles.

Some of the main SPR application areas have been summarized in [93]. This technique has been tested for concentration measurement of immunoglobulins, C-reactive protein and fibrinogen in Ref. [89]. Nanomaterial enhanced PSPR sensors for detection of DNA, proteins and hormones are discussed in [90] by measuring the SPR angle shift. SPR has been used to characterize carotenoid-binding proteins in [119], helpful in understanding the protective effects of lutein and zeaxanthin against eye diseases. Myelodysplastic syndrome (MDS) is a hematopoietic stem cell disorder and has propensity to transform to acute myeloid leukemia (AML). Vascular endothelial growth factor receptor 1 (sVEGFR-1) is a biomarker for MDS and its detection has been performed using SPR in [120] achieving a detection limit of 25 ng/mL. Another surface plasmon resonance based biosensor is demonstrated in [121] for detection of Epstein-Barr virus, which is a common virus attacking human immune system. Lable-free affinity biosensors have a drawback of not being able to distinguish between specific signal caused by analyte binding and the interfering signal due to non-specific adsorption on sensing surface, known as fouling. Thus, a antifouling layer of poly[oligo(ethylene glycol) methacrylate] may be coated on the sensor surface followed by bioreceptors to detect anti-EBV antibodies in the blood serum. A shift in resonant wavelength was observed after attachment of anti-EBV antibodies to bio-receptors and was found to be roughly proportional to the mass attached on the surface.

SPR imaging has been established as an advanced form of SPR for binding analysis [122]. Detection of small molecules via SPR imaging is a challenge due to low molecular weight and consequently low response signal. Limited immobilization capacity of the sensor is another constraint, which is enhanced by 10-fold in Ref. [123] by using dextran hydrogel coated gold sensor, leading to adequate response signals. This was used in biotin detection through streptavidin immobilized on dextran surface and reached 435 RU while no response was observed on bare gold surface.

#### 5.2.5. Surface Enhanced Raman Scattering

Raman signals are inherently weak on using visible light excitation and a low number of scattered photons are available for detection. Surface enhanced Raman Scattering is a technique by which the intensity of vibration spectra of a molecule is enhanced by several order of magnitude when it is in close proximity to nanoparticles made of gold or silver [124,125] which resonantly drives the surface charges creating a localized plasmonic light field (LSPR) enabling detection at low concentrations. In most of in-vitro SERS platforms, these nano-roughened substrates are fabricated on planar platforms like glass/silicon while for in-vivo sensing platforms SERS active surface must be fabricated on optical fibers [126] and to further enhance the sensitivity hollow core photonic crystal fiber (HCPCF) has been proposed which incorporates metallic nanoparticles and liquid analytes in air holes [127]. This suffers from shortcomings such as easy degradation and limited biocompatibility. Alternatively, SERS-nanotag labeling can be used where signals are detected from SERS probe via target biomolecule-receptor ligand interaction. However this too has disadvantages such as false positives. The technique proposed in Ref. [128] as explained later overcomes these demerits.

**Figure 26:**
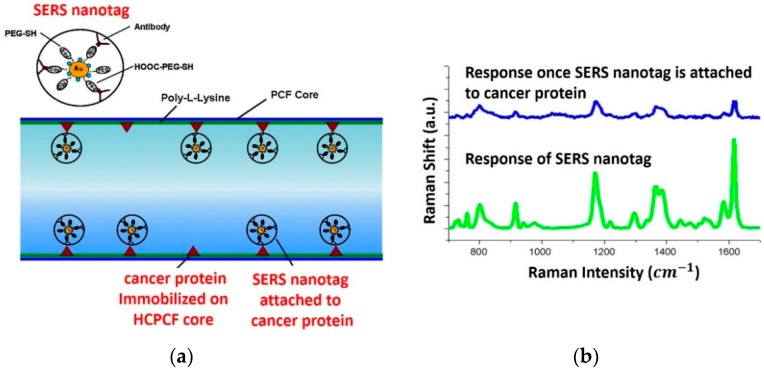
(**a**) Detection of cancer protein through anti-EGFR antibody conjugated SERS nanotag. (**b**) SERS spectra of pure anti-EGFR antibody conjugated SERS nanotag (green) and after reaction with cancer protein (blue). Reproduced from Ref. [124] with permission from Elsevier.

Photonic crystal fiber based on surface enhanced raman scattering is used for detection of cancer proteins in very low sample volume [124]. This method detects proteins at concentrations as low as 100 pg in a sample volume of 10 nL. Epidermal growth factor receptors (EGFRs) from human cancer cells are immobilized on the inner walls of HCPCF detected using anti-EGFR antibody conjugated to the SERS-nanotag which enhances the raman intensity of the antigen-antibody interaction (Figure 26). SERS-nanotag is a probe prepared by anchoring Raman active molecules on surface of gold nanoparticles conjugated with bioreceptors for specific targeting. The SERS-nanotag can alternatively be replaced with conventionally used fluorophores as done in Ref. [124] for breast cancer protein sensing. However, SERS-nanotag possess several advantages over fluorophores such as resistance to photobleaching and multiplex detection due to spectral fingerprinting [129].

Investigations of single molecules can be achieved with concentrated EM field at nano-scale hotspots. In case of SERS systems using metallic nanoparticles, the hotspots are sparse and only a small fraction of molecules end up in the nano-scale hotspots taking a long time for detection. A single molecule detection of dopamine and serotonin for understanding brain functions is proposed in Ref. [32] using a graphene-gold nanopyramid platform. While gold nanoparticles boosts sensitivity with a surface enhanced Raman spectroscopic enhancement factor of $10^{10}$, graphene adds selectivity. Summary of both label and label-free SERS methods for detection of bacteria are given in Ref. [130].

#### 5.2.6. Reflectometric Interference

**Figure 27:**
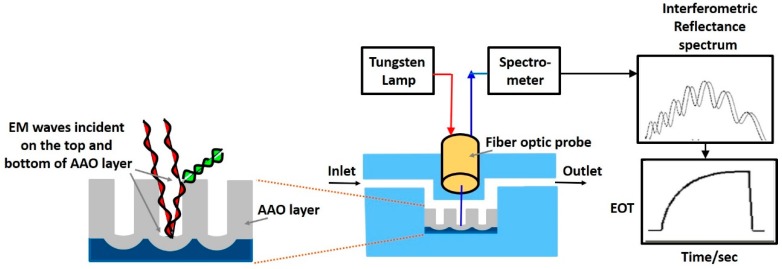
EM waves are incident on the top and bottom AAO layer and sample solution is passed from the inlet. The reflected signal from the AAO layer is processed to trace the changes in permittivity which are reflected in the interferometric reflectance spectrum [132].

Another modality using highly ordered nanoporous anodic aluminium oxide layer (AAO layer) can be used for sensing permittivity change. White light is incident at the top and bottom of the AAO layer, the reflected beams form a characteristic interference pattern which is utilized for sensing purpose and can be mapped to changes in permittivity on its surface (Figure 27). 

The pattern consists of Fabry-Perot fringes that depend on refractive index on the AAO surface and its thickness, given by a metric known as effective optical thickness (EOT). This technique is known as reflectometric interference [131]. AAO layer can be immobilized by a bioreceptor to bind to a specific target analyte which changes the wavelength of the fringe pattern along with its intensity hence is sensitive to subtle changes on its surface. Biotin-EpCAM was immobilized on the surface of the AAO layer for detection of circulating tumor cells (CTCs) in [132] by observing the changes in the Fabry-Perot interference fringes.

#### 5.2.7. Fluorescence

Fluorescence is a widely used detection method due to its selectivity and sensitivity. There are three types of fluorescent sensing- direct, indirect (here direct and indirect are in terms of fluorescence, different from what was discussed earlier) and FRET based. In direct sensing, a specific molecule is detected, by observing the fluorescence before and after a chemical reaction. In indirect sensing, a dye is used as a fluorescent tag to reflect the presence of a specific target. Finally, fluorescent energy transfer (FRET) can be used, where one fluorophore’s emission wavelength overlaps the other fluorophores excitation wavelength. In FRET, excitation of one of the fluorophore’s stimulates fluorescence in the other. A cell array biosensor was proposed containing live bacteria for mercury detection [43], accomplished by immobilizing bacterial cells on an optical imaging fiber consisting of an array of microwells. Each well containing a genetically modified Escherichia coli single bacterium fused with enhanced cyan fluorescent protein (ECFP) to identify mercury.

#### 5.2.8. Ellipsometry

Ellipsometry is an optical detection method which measures the change in polarization of light after reflection from the sample. The state of polarization is described in terms of two ellipsometric parameters i.e., ratio of Fresnel reflection amplitudes and phase shift between p- and s-polarizations. Total internal reflection ellipsometry (TIRE) is an advanced form of ellipsometry combining the advantages of ellipsometry and SPR such as high sensitivity [133]. A serum tumor marker decection based on TIRE is proposed in [134].

### 5.3. Discussions

On incidence of light in the visible or IR range, the desired bioresponse can be sensed by observing the intrinsic optical properties or changes in the optical properties such as absorption, reflectance, emission or change in interference pattern. This bioresponse usually corresponds to either observing the backscattered light (in case of direct sensors) or the affinity binding process (in case of indirect optical sensors), where the immobilized receptors on the sensor’s surface bind to the target analyte. These affinity binding biosensors can be further divided into immunosensors (antigen-antibody reaction), nucleic acid biosensors (oligonucleotide interaction with its complement) and biosensors based on ligand-bioreceptor interactions. Immobilization is extensively employed in biosensors and offers several advantages. It allows reusability of the sensor and enables continuous sensing of analytes. Microfluidic based optical sensors are an alternative to affinity binding sensors and are extensively used in POCT devices.

The other important classification is based on whether the sensors employ in vivo or ex vivo technique for measurements. PPG modality (in vivo) is commonly employed to obtain Sp$O_{2}$ and other vital information. The choice of wavelength for light-tissue interactions is important. Since PPG is based on measuring the reflected/backscattered light from the tissues, it is worthwhile to choose a wavelength which is not strongly absorbed by tissue. Biological tissue shows strong absorption in the UV range (by melanin) and longer infrared region however, allows visible red and infrared light to pass more easily, i.e., poor absorber of red light allowing light to penetrate much deeper, where large vascular and tissue beds are present [101]. PPG signal not only contains backscattered light through the tissue but also artifacts from ambient light and from subject’s skin structure/pigments. Green light has much greater absorptivity for oxyhaemoglobin and deoxyhaemoglobin compared to IR, therefore change in reflected green light is greater than reflected infrared signals giving better signal to noise ratio. However, depth of penetration is limited and hence can only be used where blood perfusion is more. Whereas, red and IR are better for deep tissue blood flow measurements. Reflectance mode PPG is advantageous in terms of flexibility in sensor placement position, however is highly susceptible to motion artifacts and pressure disturbances.

Optical biosensors have also been widely used to analyse biomolecular interactions (ex vivo) including enzyme-substrate, antigen-antibody and DNA-DNA hybridizations. Conventional optical sensors were based on fiber-optics, interferometers, fluorescence spectroscopy and surface plasmons. Recent trends in optical biosensor development are mainly focused on incorporating noble metal nanoparticles, for instance, local surface plasmon resonance and surface enhanced raman scattering. These are noble metal nanostructures such as gold and silver have been employed for enhancing PSPR performance [90] through additional LSPR. In comparison with surface plasmon polaritons (SPPs), local surface polaritons (LSPs) generate an electromagnetic field which is more closely tied to the surface of the metal and facilitates even more localized probing at the interfaces, on a scale comparable to the dimension of biomolecules. Although LSP-based sensors [121] prove advantageous for monitoring interactions of low numbers of biomolecules, however, they are limited by their bioanalytical applications due to limited number of molecular binding events measured by the sensor. Though these nanometals enhance sensitivity, they are toxic to humans.

Another noteworthy recent development is the utilization of quantum dots over the conventional methods of using fluorescent dyes/proteins for biodetection. Its merits over fluorophores are broad absorption spectrum, long excited state fluorescent lifetimes and resistance to photobleaching. Another important feature is simultaneous detection of multiple signals which is hard to achieve in FRET fluorophores due to overlapping of absorption and emission spectra [135]. Thus, QDs are envisioned to create a new generation of robust biosensors.

Using photonic band gap material instead of metal films in sensor fabrication is another recent development. As seen earlier, one biosensing modality in optical biosensors is through excitation of surface waves at the interface of two different dielectric mediums through EM waves which evanescently decays away from the interface and is extremely sensitive to changes in permittivity at the interface. This is seen to be possible in both metal-dielectric interface and photonic band gap film-dielectric interface. The advantages of using photonic band gap films over metal film as medium for surface waves are: sharper resonance leading to enhanced sensitivity [136], ability to be engineered to operate at any wavelength, which is not possible with metal films [87], and greater mechanical and chemical robustness than metal films. Another advantage of photonic crystal surface waves over surface waves propagating in metal is that both p-polarized and s-polarized optical surface waves can be used for sensor applications [115]. Table 3, Table 4, Table 5, Table 6, Table 7 and Table 8 list the various EM wave biosensors categorized on the basis of direct and indirect biosensing methods (described in Section 3), labelled and label-free sensing, in vivo and ex vivo measuring techniques, their interaction modality with EM waves, their detection methods and their applications. Some common applications along with biomatter-wave and detection modalities of some popular IR and visible biosensors are listed in Table 7 and Table 8.

## 6. Characteristics and Safety Aspects of EM Waves

Having studied about the various EM wave sensors and their interaction modalities with living organisms, it is essential to note its adverse effects on the organism as well. Following are some of the noteworthy properties and drawbacks of exposing the living organism with EM waves.

The wavelength of EM waves (λ) is significantly reduced in tissues due to high dielectric constant of biological tissue. Considering K to be the propagation constant for power transmission through biological tissue and $K_{0}$ to be the propagation constant of free space, then $K=K_{0}{\left(\frac{\epsilon^{*}}{\epsilon_{0}}\right)}^{1/2}=\beta -j\alpha$, and $\lambda =\frac{2\pi }{\beta }$. Along with a large reduction in wavelength, there is also a large absorption of energy in tissue which can result in heating [2].

In this paper, we talk about non-ionizing radiations which cannot ionize atoms due to their small energy content. UV light is the most energy rich form of non-ionizing radiation, and in rich doses can cause eye inflammations and sunburns [140]. Visible light energy is absorbed by retina cells and converted to nerve impulses enabling us to see. However, high intensity laser light may cause irreparable damage to eye cells through burns [141]. Blue light having high energy content is capable of causing harmful photochemical reactions to light sensitive eye cells even at low intensities. IR energy is converted to heat in the body and is absorbed mostly by skin due to skin effect discussed later [2]. Radio waves also cause a heating effect in the body, however, the effect is deeper. Electromagnetic fields of extremely low frequency (up to a few kHz) are capable of producing strong currents which can stimulate nerves and muscles [142,143].

In general, the maximum power absorbed by the body occurs at a certain ratio of the body dimensions to the wavelength. For instance, when the long body axis is parallel to the field, the ratio of body length to wavelength is 0.4, while in other orientations it is approximately 1 [142]. RF power interaction with biological tissues causes ionic conduction and vibration of dipoles molecules (dipolar polarization) producing a heating effect from the dielectric loss at relaxation frequency. This heating effect is either compensated by thermoregulatory action or accompanied by a local or overall increase in temperature. Non-uniform heating may result in formation of hotspots which may cause a number of secondary effects such as microwave hearing, i.e., hissing sounds [144]. The average power density to induce this effect is 16 mJ/kg.

Apart from heating, non-thermal effects of RF wave interaction with human body have been observed. An alternating electric field of few V/cm applied perpendicularly to a nerve cell membrane, can form a potential of few mV across the membrane which is sufficient to excite the nerve @1 MHz. This potential decreases with increase in frequency [143]. Studies at low frequency radio fields of 5–25 Hz having low intensity have suggested changes in transmembrane flow of calcium ions [145] and signaling mechanisms that involve membrane receptors [146]. Exposure to low-level pulsed microwaves have been reported to affect brain neurochemistry and response to stress [147,148]. Another non-thermal effect of RF waves is pearl chain effect [149], where particles like blood cells and starch when placed in RF field (1–100 MHz) form chains parallel to the electric field. However, these forces are not significant unless large intensity fields of few 100V/cm are applied. Dielectric saturation [2] is also possible, occurring in solution of proteins and other macromolecules due to intense fields where polarized sidechains of macromolecules align with the field, leading to a possible breakage of hydrogen bonds.

Another important phenomenon occurs when a biological tissue is exposed to EM field, known as skin effect [4] in which fields, currents and charges concentrate near the surface. The depth upto which the currents are concentrated is known as skin depth and it decreases with increase in frequency. It varies from a fraction of mm at >100 GHz to a few cm at few GHz, in high water content tissues (more lossy). In low water content tissues (less lossy), it can go up to a meter at few MHz [18]. Higher the frequency, the smaller is the penetration hence internal organs are more protected than at low frequencies.

## 7. Conclusions

The nature of interaction of EM waves with biomatter provides tremendous opportunities in the field of biosensing. Biomatter behaves as a dielectric, hence any changes are traced in terms of dielectric properties; permittivity and conductivity. Dielectric signatures (permittivity vs frequency) can be obtained either in a broadband fashion (over a wide frequency range) or in cases when that broad spectrum of information is of little use, permittivity changes only over a narrowband of frequencies can be obtained. The choice of frequency band is based on whether intracellular information is needed or not, if so frequencies in the microwave region are suitable as most of the dipolar relaxations experienced by water in tissues occurs in this region. On application of an alternating electric field on a cell in suspension medium, several dispersions are observed. Though the dispersions vary in different biological tissues, in general, we can say that α-dispersion is observed first, in the kHz range. β-dispersion is observed next in the MHz range. Both of these dispersions are sensitive to changes in cell membrane potential and thickness. Finally, γ-dispersion is observed at GHz frequencies and is sensitive to changes in intracellular content (e.g., water).

In this paper, we summarized the different optical, microwave, millimeter wave, radio frequency and terahertz biosensor modalities. Sensing is realized in two ways; either by directly observing the intrinsic EM wave properties or indirectly by measuring the change in EM wave properties. In direct optical sensors, the backscattered light from the arteries can be used to obtain information about vital signs such as heart rate, whereas in indirect optical sensors, change in concentration on biochemical reaction (affinity binding) is detected by observing changes in optical properties such as fluorescence, refection intensity, photoluminescence, interference pattern or refractive index. In direct RF, mmW, MW and THz biosensors, the cell’s dielectric signature is used to distinguish between cancerous and healthy cells. Motion detection, as in case of Doppler to obtain vital sign information from the periodic chest wall movement, is also possible. A more popular technique is that of proximity coupling alternatively known as near field sensing, where the periodic movement is sensed by the near field of the resonator as a variable capacitance. In indirect sensing, permittivity change is sensed. Here, mostly capacitive sensing is employed, whose capacitance is a function of permittivity. This variable capacitance is placed in an oscillator circuit and changes in resonant frequency and scattering parameters are detected.

The different application areas for EM wave sensors are highlighted in this paper. These include detection of cancer, tumor cells, vitamins (biotin), neurotransmitters (serotonin), pathogens (bacteria), DNA, heart rate, Sp$O_{2}$ and other vital signs such as cardiac output and respiration. All in all, RF, mmW, MW, THz and optical biosensors have their merits and demerits. Optical sensing has been in use for a long time and RF/mmW/MW/THz biosensors are relatively new, and substantial research needs to be done to establish them as a standard approach. Point of care diagnostics are dominated by electrochemical and optical based sensors, however, usually require samples of blood or plasma and gets very difficult for continuous monitoring. Though large amount of research has gone into the development of RF biosensors, there are yet no RF biosensors available in the market except those for vital sign detection based on doppler’s principle and a blood glucose monitoring device named Glucowise. One major challenge faced in the reproducibility of microwave senors in the market, is that microwave signals are weak and difficult to detect. Commercializing these RF biosensors will be greatly beneficial as they will pave way for non-invasive sensing and facilitate continuous monitoring applications.

## Figures and Tables

**Table 1:** Dispersion characteristics and its applications.

Frequency Range	What Can Be Sensed	Application
α and β-dispersion	Changes in cell membrane potential, Changes in cell membrane thickness, Cells with different cell shape can be distinguished	Characterizing the different types of WBCs (e.g., lymphocytes, neutrophils and monocytes) [19], distinguish dead cells from living cells [12]
γ-dispersion	Intracellular properties and contents	Detection of biomolecules e.g., glucose [21], biomarkers for cancer cells [8]

**Table 2:** Commercially available biosensors [25,26].

Type of Biosensor	Manufacturer	Principle	Sample Required
Glucose	Glucowise	Millimeter wave spectroscopy	No blood sample required
Glucose	Bayer, Nipro Diagnostics, Prodigy Diabetes Care, Aga Matrix, Simple Diagnostics	Electrochemical-cassette and strips	Blood sample required
Glucose	EKF Diagnostics, Maxim Integrated	Optical-reflectometry test strips	Blood sample required
hCG pregnancy test	Sofia	Optical fluorescent test	Urine sample
hCG pregnancy test	Alere, Coretests	Electrochemical-cassette and strips	Urine sample
HIV	TFS biosensor fluorometers	Optical-fluorescence test	Blood sample
HIV	CLUNGENE	Electrochemical-strips and cassettes	serum/plasma/whole blood
Malaria	CLUNGENE, Egens Diagnostics, Access Bio, Inc., Alere	Electrochemical-strips and cassettes	Serum/plasma/whole blood
Tuberculosis	CLUNGENE, Alere, Standard Diagnostics, Inc., United Surgical & Diagnostics	Electrochemical-strips and cassettes	Serum/plasma/whole blood
Carcinoembryonic Antigen (CEA) Rapid Diagnostic Test	CLUNGENE, Run Bio Tech Co. Ltd.	Electrochemical-strips and cassettes	Serum/plasma/whole blood
Prostate Specific Antigen (PSA) Rapid Diagnostic Test	CLUNGENE, Run Bio Tech Co. Ltd.	Electrochemical-strips and cassettes	Serum/plasma/whole blood
Prostate Specific Antigen (PSA) Rapid Diagnostic Test	CLUNGENE, Run Bio Tech Co. Ltd.	Electrochemical-strips and cassettes	Serum/plasma/whole blood
Cardiac markers- troponin	CLUNGENE	Electrochemical-strips and cassettes	Serum/plasma/whole blood
Monitor biomolecular interactions in real time	Biacore	Optical-SPR+affinity binding	Serum/plasma/whole blood
Monitor biomolecular interactions in real time	Affinity biosensors	Optical- evanescent waves	Serum/plasma/whole blood
Identification of pathogens-bacteria and virus	IBIS Technologies	Optical- SPR+affinity binding	Serum/plasma/whole blood
Heart rate	ELCAT Medical Systems, Shimmer Sensing, Gutmann MD	Optical - photoplethesmography	No sample required
Fetal Heart rate	Laerdal	RF-doppler	No sample required

**Table 3:** RF biosensors.

Paper	Operating Range (Hz)	In Vivo/Ex Vivo	Label/Label Free	Direct/Indirect	Biomatter Wave Modality	Detection Modality	Application
[11]	1–40 G	ex vivo	Label-free	Direct	CPW transmission	Attenuation constant and complex permittivity	Dielectric characterization of cancer cells
[71]	915 M	ex vivo	Label	Indirect	Effective length of antenna is increased with increase in target concentration using affinity binding	Interrogation distance	Concentration detection of rabbit IgG
[51]	3 G	in vivo	Label-free	Direct	Circulator resonator using near field	Oscillation frequency deviation converted to a Voltage signal by PLL	Vital sign detection
[7]	2 G	ex vivo	Label-free	Indirect	SRR using affinity binding	S-parameters/change in resonance frequency	Detection of heparin
[21]	2 G	ex vivo	Label-free	Indirect	SRR using affinity binding	S-parameters/change in resonance frequency	Detection of glucose
[52,53]	2.4 G	in vivo	Label-free	Direct	Array resonator using near field	S-parameters	Pulse detection
[8]	2 G	ex vivo	Label-free	Indirect	Hairpin resonator using microfluidic channel	S-parameters	Detection of melanoma
[54]	360 M	in vivo	Label-free	Direct	Planar resonator using near field	PLL transforms the frequency change to voltage variation	Vital sign detection
[43]	2 G	in vivo	Label-free	Direct	Harmonic Doppler radar using heterodyne Rx	Phase modulated signal	Vital sign detection/Cardiopulmonary monitoring
[44,81]	2.4–3 G	in vivo	Label-free	Direct	Doppler (self-injection locking VCO)	Phase modulated signal	Vital sign detection
[41]	2.5 G	in vivo	Label-free	Direct	Differential front end Doppler	Phase modulated signal	Vital sign detection
[42]	2.5 G	in vivo	Label-free	Direct	Doppler (mutual injection locking)	Phase modulated signal	Vital sign detection
[57]	180 M	ex vivo	Label-free	Indirect	SRR using affinity binding	S-parameter/resonant frequency shift	Biomolecular detection
[82]	40 k	ex vivo	Label-free	Indirect	Resonator mass sensor using affinity binding	S-parameter/resonant frequency shift	Biomolecular detection
[83]	-	in vivo	Label-free	Direct	Strain sensor	S-parameter/resonant frequency shift	Helpful in fractured patients

**Table 4:** Microwave biosensors.

Paper	Operating Range (Hz)	In Vivo/Ex Vivo	Label/Label Free	Direct/Indirect	Biomatter Wave Modality	Detection Modality	Application
[37,38]	5.8 G	in vivo	Label-free	Direct	Doppler	Phase modulated signal	Vital sign detection
[45]	26–40 G	in vivo	Label-free	Direct	ultra-wideband (UWB) radar	Phase modulated signal	Vital sign detection
[69]	12 G	ex vivo	Label-free	Indirect	Interdigital capacitance sensor using microfluidic channels	PLL Demodulator gives voltage output signal	Rapid particle counting and single particle sensing
[16]	5 G	ex vivo	Label-free	Indirect	Microwave cavity sensor using microfluidic channels	S-parameter/resonant frequency shift	Pig blood d-glucose
[62]	28 G	ex vivo	Label-free	Indirect	Microstrip based stub as capacitive sensor using microfluidic channels	Shift in oscillation frequency and output power	Malignant cell growth investigation, cell cultivation monitoring
[36]	13 G	ex vivo	Label-free	Indirect	Microstrip open stub as capacitive sensor using microfluidic channels	S-parameters/change in resonance frequency	Detection of Fibroblast cells
[63]	29 G	ex vivo	Label-free	Indirect	Coplanar transmission line using microfluidic channels	Shift in oscillation frequency	Discrimination of fat and calcium in blood
[65]	25 G	ex vivo	Label-free	Direct	UWB planar antenna	Relative permittivity	Cell quantification and counting in solution
[46]	4–10 G	in vivo	Label-free	Indirect	Split-ring resonator (SRR) using affinity binding	Conduction and specific absorption rate	Detection of tumors in human breast tissues
[58]	12 G	ex vivo	Label-free	Indirect	SRR using affinity binding	S-parameters/change in resonance frequency	DNA sensing
[59]	10 G	ex vivo	Label-free	Indirect	SRR using affinity binding	S-parameters/change in resonance frequency	Biotin and streptavidin sensing
[64]	10 k–8 G	ex vivo	Label-free	Indirect	Coplanar waveguide using affinity binding	S-parameters/change in resonance frequency	DNA biosensor
[67]	19.2–20.8 G	ex vivo	Label-free	Indirect	Interdigital capacitor (IDC) embedded in VCO using microfluidic channels	PLL gives output voltage signal	Characterization of liquids
[70]	5–14 G	ex vivo	Label-free	Indirect	IDC using microfluidic channels	PLL gives output voltage signal	Discrimination of colorectal cancer

**Table 5:** Millimeter wave.

Paper	Operating Range (Hz)	In Vivo/Ex Vivo	Label/Label Free	Direct/Indirect	Biomatter Wave Modality	Detection Modality	Application
[34]	40 G	ex vivo	Label-free	Direct	CPW transmission line	S-parameter/resonant frequency shift	Identifying cancer cells
[61]	35 G	ex vivo	Label-free	Direct	WGM resonator	Inverse quality factor and resonant frequency shift	Nanoliter liquid characterization
[35]	G–THz	ex vivo	Label-free	Indirect	Resonator using affinity binding	S-parameter/resonant frequency shift	Biomolecular detection, glucose detection and hyperthermia treatment

**Table 6:** Terahertz biosensors.

Paper	Operating Range (Hz)	In Vivo/Ex Vivo	Label/Label Free	Direct/Indirect	Biomatter Wave Modality	Detection Modality	Application
[72]	0.4–1 T	ex vivo	Label-free	Indirect	Resonator using microfluidic channels	S-parameter/resonant frequency shift	Liver cancer biomarker detection
[75]	0.3–0.8 T	ex vivo	Label-free	Indirect	Planar resonator	S-parameter/resonant frequency shift	Detection of DNA hybridization

**Table 7:** Infrared Biosensors.

Paper	Operating Range (nm)	In Vivo/Ex Vivo	Label/Label Free	Direct/Indirect	Biomatter Wave Modality	Detection Modality	Application
[127]	200–900 nm	in vivo	Label-free	Indirect	Near infrared spectroscopy	Backscattered light	Detection of neural activity
[117]	400–800	ex vivo	Label-free	Indirect	LSPR using affinity binding	Shift in the extinction maximum in LSPR spectrum	Detection of serum human epididymis secretory protein in patients with ovarian cancer
[118]	750	ex vivo	Label-free	Indirect	LSPR using affinity binding	Change in reflected beam’s elliptical polarization parameters	Detection of short DNA molecules
[121]	780	ex vivo	Label-free	Indirect	SPR using affinity binding	Shift in resonant wavelength	Diagnosis of Epstein–Barr virus infection
[97]	730–880	in vivo	Label-free	Direct	Backscattering	PPG signals	Heart rate and Sp$O_{2}$

**Table 8:** Visible light Biosensors.

Paper	Operating Range (nm)	In Vivo/Ex Vivo	Label/Label Free	Direct/Indirect	Biomatter Wave Modality	Detection Modality	Application
[86]	365	ex vivo	label	Indirect	QD using affinity binding	Photoluminesc-ence	Detection of cancer biomarker
[87]	470	ex vivo	Label-free	Indirect	PC SW using affinity binding	Reflectivity	Detection of Bovine Serum Albumin
[115]	658	ex vivo	Label-free	Indirect	PC SW using affinity binding	Refractive index and adlayer thickness	Detection of vitamin-biotin
[88]	670	ex vivo	Label-free	Indirect	Surface waves using affinity binding	Interferometry	Detection of avian influenza
[137]	400–800	in vivo	Label-free	Direct	Laser Doppler flowmetry	Number of moving cells and their velocity	Microcirculation Changes in tissue
[124]	633	ex vivo	Label	Indirect	SERS Photonic crystal fiber using affinity binding	SERS spectrum	Detection of cancer proteins
[89]	250–800	ex vivo	Label-free	Indirect	LSPR using affinity binding	Absorption Spectra	Detection of immunoglobulins, C-reactive protein and fibrinogen
[132]	400–900	ex vivo	Label-free	Indirect	Reflectometric interference using affinity binding	EOT	Detection of circulating tumor cells
[90]	540–780	ex vivo	Label-free	Indirect	SPR using affinity binding	SPR angle shift	Detection of olegonucleotides, proteins and hormones
[123]	365	ex vivo	Label-free	Indirect	SPR imaging sensor using affinity binding	Total response signal in RU (response units)	Probing small molecule binding events
[138]	400–500	ex vivo	Label-free	Indirect	SPR using affinity binding	Total response signal in RU (response units)	Characterization of Protein-Carotenoid Interactions
[93]	600–850	ex vivo	Label-free	Indirect	SPR using affinity binding	Total response signal in RU (response units)	Antigen-antibody and protein-DNA interaction detection
[120]	-	ex vivo	Label-free	Indirect	SPR using affinity binding	Total response signal in RU (response units)	Myelodysplastic syndrome biomarker (VEGFR-1) detection
[92]	440–480	ex vivo	Label	Direct	Genetically modified bacterium	Fluorescence	Mercury detection
[134]	633	ex vivo	Label	Indirect	Total internal reflection imaging ellipsometry biosensor using affinity binding	Charge coupled device (ccd) camera stored in grayscale format	Serum tumor marker detection
[91]	660–850	in vivo	Label-free	Direct	Backscattering	PPG signals	Heart rate and Sp$O_{2}$
[139]	660–940	in vivo	Label-free	Direct	Backscattering	PPG signals	Heart rate and Sp$O_{2}$
